# Receipt of Medicines Information From the Internet and Other Information Sources Among Adult Medicine Users in Developed Economies, 2010-2025: Systematic Review

**DOI:** 10.2196/71984

**Published:** 2026-05-20

**Authors:** Niina Mononen, Milla-Maria Salo, Daria Timonen, Marika Pohjanoksa-Mäntylä, Marja Airaksinen

**Affiliations:** 1 Clinical Pharmacy Group, Division of Pharmacology and Pharmacotherapy, Faculty of Pharmacy, University of Helsinki, Viikinkaari 5, Helsinki, 00014, Finland, 358 0504480780

**Keywords:** medicines information, medicines information source, receipt of medicines information, internet, medicine user, systematic review

## Abstract

**Background:**

The internet, social media, and digital health tools have transformed access and receipt of medicines information (MI), complementing or replacing traditional sources, such as physicians, pharmacists, and package leaflets.

**Objective:**

This study aimed to examine the prevalence and patterns of adult medicine users’ receipt of MI from electronic sources (eg, internet and social media) compared with traditional sources, and to assess trends in the receipt of MI in developed economies since 2010.

**Methods:**

A systematic search was conducted in CINAHL, the Cochrane Library, ProQuest, Scopus (including Embase and MEDLINE), and Web of Science databases for peer-reviewed studies conducted and published between January 1, 2010, and December 31, 2025. Studies including adults (≥18 years) in developed economies were eligible. The review followed PRISMA (Preferred Reporting Items for Systematic Reviews and Meta-Analyses) 2020 guidelines. Methodological quality and risk of bias were assessed using the Mixed Methods Appraisal Tool. Due to substantial heterogeneity, findings were synthesized narratively without meta-analysis.

**Results:**

Twenty-six studies from 10 countries, involving 19,891 medicine users, were included. Most studies were cross-sectional surveys (n=19), with only one being a national long-term trend study. Physicians, pharmacists, and package leaflets consistently remained the most common MI sources, irrespective of patient group, country, or study design. No clear temporal trends in MI receipt were observed. Variations in the frequency of MI receipt were evident across research methods, medicine user groups, and types of medicines. Individuals with greater familiarity with the internet were more likely to receive MI from electronic sources, such as websites and social media. In most studies, MI obtained from social media was not distinguished from other internet-based sources. Half of the studies focused on heterogeneous medicine user groups (n=14), while the remainder examined specific medicine user groups (n=12). Although the methodological quality of the studies was generally acceptable, only 3 studies reported the use of an explicit theoretical framework.

**Conclusions:**

Traditional MI sources remain central for adult medicine users despite the growing role of electronic platforms. While the receipt of MI from electronic sources appears more common among internet-experienced users, no significant temporal trends in using these sources were identified. Further research is needed to better distinguish between different digital MI sources, including artificial intelligence–based MI sources, and to explore their evolving roles, implications for health-related decision-making, and associations with user characteristics and health contexts in the digital era. This review is innovative in systematically comparing traditional and electronic MI sources across developed economies and in highlighting methodological and conceptual gaps in existing research. By synthesizing cross-national evidence and identifying the need for clearer differentiation of digital MI subtypes, the study contributes to the field and informs the development of more targeted, reliable, and user-centered MI strategies in real-world health care settings.

## Introduction

Medicines information (MI) has become increasingly accessible to consumers and medicine users through multiple modalities, reflecting decades of regulatory, technological, and professional developments [1-3]. Major drug safety crises, particularly the thalidomide catastrophe in the early 1960s, demonstrated the public health consequences of limited availability of MI, leading to major regulatory reforms [1,2,4-14]. The reforms included the introduction of statutory written medicines information (WMI) to consumers, such as standardized package leaflets (PLs) in the European Union (EU) [2,8,10-12]. The thalidomide crisis, followed by other drug safety crises, was a driving force for risk management measures, including a global shift toward empowerment and patient self-management [3,15,16]. This positioned physicians, pharmacists, and other health care professionals (HCPs) on the frontline as MI providers [2,16,17]

Since the 1990s, the digital transformation of society has profoundly reshaped access to health information and MI [2,18-20]. Online sources now range from official regulatory databases and health care provider websites to commercial platforms, patient communities, social media, and mobile health apps [21-23]. Over the past decade, and particularly following the COVID-19 pandemic, digital health services have expanded rapidly, including telehealth consultations, remote medication counseling, chat-based pharmacy services, and electronically provided PLs [16]. More recently, artificial intelligence (AI)-based tools and generative language models have emerged as novel channels for health- and medicines-related information seeking. These developments have enabled more immediate, interactive, and personalized forms of receiving MI, potentially supporting patient empowerment in self-management of chronic conditions and mitigating minor symptoms [24]. At the same time, concerns have been raised regarding information quality, misinformation, digital health literacy, and unequal access across population groups [25].

A growing body of research has examined online health information-seeking behaviors and digital health interventions [18,19,26]. Fewer studies have focused specifically on MI as a distinct domain. Existing research mainly addresses single information channels in isolation, such as internet use or social media, without systematically comparing electronic sources with traditional sources, including physicians, pharmacists, and PLs. Furthermore, it remains unclear whether the receipt of internet-based MI has increased over time relative to traditional modalities. Therefore, a comprehensive synthesis examining patterns of MI receipt across electronic and traditional sources is warranted. Understanding how adult medicine users receive MI is of considerable public health and clinical importance. In the context of rapid digital transformation, synthesizing evidence on the relative use of electronic and traditional MI sources is essential to inform policy, health care practice, and future research.

This systematic review aimed to examine the prevalence and patterns of the receipt of MI from electronic and traditional sources among adult medicine users in developed economies since 2010. The specific objectives were (1) to compare the frequency of receipt of MI from electronic sources (eg, internet and social media) and traditional sources (eg, physicians, pharmacists, and PLs) and (2) to assess whether the receipt of internet-based MI has increased over time. In addition, the review sought to identify methodological and conceptual gaps in the existing literature to guide future research and policy development in the rapidly evolving digital health context.

## Methods

### Design

This study was conducted as a systematic review in accordance with the PRISMA (Preferred Reporting Items for Systematic Reviews and Meta-Analyses) 2020 guidelines [27]. The PRISMA 2020 flow diagram is presented in the Results section, and the completed PRISMA 2020 and PRISMA-S (Preferred Reporting Items for Systematic Reviews and Meta-Analyses–Search extension) checklists are provided in Checklists 1-3 [27,28]. The Covidence systematic review software (Veritas Health Innovation) was used to manage study selection, screening, and data extraction throughout the review process [29].

### Eligibility Criteria

Studies were eligible for inclusion if they met predefined criteria regarding the study population, interventions, outcomes, context, study design, and time, as summarized in Table 1. Eligible studies included adults aged 18 years or older who were current medicine users and examined the receipt of MI from any information source. Studies were required to be conducted in developed economies according to the United Nations classification [30], published in peer-reviewed international scientific journals in English, and published between January 1, 2010, and December 31, 2025. Both quantitative and qualitative original research studies, as well as systematic reviews and meta-analyses, were considered for inclusion. Conference abstracts, editorials, narrative reviews, doctoral and master’s theses, and gray literature were excluded.

The choice of 2010 as the cut-off year was informed by the transition of MI practices from predominantly paper-based formats to electronic formats, which became more pronounced during the 2010s [2]. In addition, the need for improved EU-level coordination of MI provision to consumers was identified in 2005, followed by the issuance of recommendations on quality and accessibility by the European Commission in 2008 [31,32]. Consequently, MI practices began to be addressed more systemically in several member states; for example, a national MI strategy was introduced in Finland in 2012 [1,2]. Furthermore, earlier studies indicated that the literature published before 2010 on medicine users’ receipt of MI was limited [2].

**Table 1.** Inclusion and exclusion criteria for the literature search based on the PICOT (Population, Intervention, Context, Outcomes, and Time) framework.

Component	Inclusion criteria	Exclusion criteria
Population (P)	Participants aged ≥18 years Current medicine users Outpatients, inpatients, or hospital-discharged patients Native population Laymen	Participants aged <18 years People not currently using medicines People using illegal substances or people with substance use (including doping use). People using only herbal medicines, complementary and alternative medicines (CAM), or dietary supplements. Immigrant population Health care professionals or health care students
Intervention (I)	MI^a^ sources actually used.	The following topics were excluded: Receipt or seeking of information about health, health care services, travel health advice, food, and nutrition information. Receipt or seeking information about someone else’s medicines (eg, children, spouse, and friend). Usability of online MI or health apps. Receipt of MI has not been clearly separated from health information. Research results of medicine users and nonusers have been processed together and not separated from each other. Receipt of MI among individuals who are illiterate or have low or limited literacy skills. Research focused on the usage or usability of only one MI source (eg, certain web pages, mobile phone apps, medical literature platforms, and so on). Use of national electronic health record systems (eg, My Kanta).
Context (C)	Study was conducted in developed economies^b^. All types of research methods. Peer-reviewed full paper. Published in English.	Study was conducted in developing economies^b^. Conference abstracts, editorials, narrative reviews, doctoral and master’s theses, and gray literature.
Outcomes (O)	Receipt of MI among the study population from any information sources (eg, physicians, pharmacists, PLs^c^, the internet, and so on).	—^e^
Time (T)	Study was conducted and published between January 1, 2010 and December 31, 2025^d^.	Study was conducted and published before 2010 or after December 31, 2025.

a MI: medicines information.

b Country selection was based on the classification by the United Nations of developed economies (ie, Australia, Austria, Belgium, Bulgaria, Canada, Croatia, Cyprus, Czech Republic, Denmark, Estonia, Finland, France, Germany, Greece, Hungary, Iceland, Ireland, Italy, Japan, Latvia, Lithuania, Luxembourg, Malta, the Netherlands, New Zealand, Norway, Poland, Portugal, Romania, Slovakia, Slovenia, Spain, Sweden, Switzerland, the United Kingdom, and the United States [30]).

c PL: package leaflet.

d Papers that did not mention the year of the study or data collection were included if the study was published in 2010 or later.

e Not available.

### Information Sources

A comprehensive literature search was conducted using 5 international bibliographic databases: CINAHL, the Cochrane Library, ProQuest, Scopus (including Embase and MEDLINE), and Web of Science. In addition to database searches, the reference lists of all included studies were screened manually to identify any additional eligible studies. The most recent search was conducted on January 15, 2026, to ensure inclusion of the most up-to-date evidence. Full search strings and database filters for each database are provided in Multimedia Appendix 1. All searches were rerun before final analysis to ensure inclusion of the most recent studies.

### Search Strategy

A systematic literature search was conducted using predefined inclusion and exclusion criteria, Boolean operators, and database-specific filters (Table 1 and Multimedia Appendix 1). One researcher (NM) performed the database searches in collaboration with an information specialist from the University of Helsinki, and the search strategy was reviewed by the research team before execution. The following five international bibliographic databases were searched: CINAHL, the Cochrane Library, ProQuest, Scopus (including Embase and MEDLINE), and Web of Science. The search strategy incorporated the following key concepts and terms: “medicines information” or “drug information”; “receipt” or “receive”; “search” or “seek”; “medicines information source or channel” or “drug information source or channel”; “consumer,” “interviewee,” “medicine user,” “respondent,” or “patient”; and “women” or “men.” Full search strings and database-specific filters are provided in Multimedia Appendix 1.

Iterative pilot searches were conducted to assess the sensitivity and relevance of the search strategy. These pilots indicated that some relevant studies referred to participants exclusively as “women” or “men,” necessitating the inclusion of these terms to ensure comprehensive identification of eligible studies. The pilot searches further demonstrated the need to refine and narrow the eligibility criteria applied to focus the review specifically on the receipt of MI among adult medicine users, which led to the exclusion of a substantial number of studies that did not meet this criterion.

In this review, “receipt of MI” was defined as the active acquisition and obtaining of information regarding medicines, whereas “seeking MI” refers solely to the act of searching for information and does not necessarily result in obtaining it.

### Selection Process

Study selection was managed using Covidence systematic review software [29], which enabled continuous monitoring of selection consistency throughout the process. All identified records were imported into Covidence. After duplicate removal, at least two researchers (NM, DT, MMS, and MA) independently screened titles and abstracts. Full texts of potentially eligible studies were independently assessed by two reviewers. Disagreements were resolved through discussion and consensus within the research team. Final inclusion decisions were made collectively by the study group, and consensus was achieved for all included studies. According to the Covidence documentation, agreement at the final stage of study selection was 100%. In addition, reference lists of all included papers were screened to identify further potentially relevant studies. All additional records identified through reference list screening that met the eligibility criteria were subsequently included in the review.

### Data Collection Process

Data extraction was conducted using a standardized extraction table developed specifically for this review. Two researchers independently extracted data, which were then reviewed and verified by the remaining members of the research team. Any discrepancies were resolved through discussion. Data management and preliminary organization were conducted using Microsoft Word and Microsoft Excel (Windows 10).

### Study Risk of Bias Assessment

The methodological quality of the included studies was assessed using the Mixed Methods Appraisal Tool (MMAT; version 2018) (Multimedia Appendix 2) [33,34]. The MMAT was selected for its capacity to evaluate a wide range of study designs, including quantitative, qualitative, and mixed methods studies. The tool comprises design-specific criteria to evaluate methodological rigor and internal validity within each study category. For example, in quantitative descriptive studies, it explicitly assesses the risk of nonresponse bias [34,35].

At least 2 reviewers (NM, MMS, and MA) independently appraised each study using the MMAT checklist. Discrepancies were resolved through discussion, and a third reviewer was consulted when necessary. Each criterion was rated as “Yes” (1 point), “No” (0 points), or “Can’t tell” (0 points), and the sum of the scores indicates the total quality of each study. Screening questions (S1 and S2) were excluded from the total scores. For mixed methods studies, the overall quality score was determined by the lowest score of the individual component, as the overall quality of a combination cannot exceed the quality of its weakest component [33,35]. Risk of bias assessments informed the interpretation of findings but were not used as exclusion criteria. Final quality ratings were determined by consensus among the reviewers.

### Data Extraction

Data were extracted using a standardized extraction table developed specifically to address the objectives of this systematic review. Studies were content analyzed and categorized, with quantitative and qualitative studies analyzed and reported separately. Two researchers independently performed data extraction, and the results were subsequently reviewed and approved by the remaining members of the research team. Discrepancies were resolved through discussion to ensure consistency and accuracy. Data management and organization were conducted using Microsoft Word and Microsoft Excel (Windows 10), with the analysis proceeding in sequential stages.

Extracted study characteristics included authorship, year of publication, country, aim of the study, study design, study setting, study population, medicine user group, MI sources investigated, and key findings related to the receipt of MI. Where reported, additional methodological and contextual information, such as recruitment and sampling methods, definitions and measurement of the receipt of MI, outcome measures, data collection period, analytical approaches, theoretical frameworks, and quality and risk-of-bias assessments, were also collected to support transparent reporting and facilitate interpretation of heterogeneity across studies.

Tables2  3 summarize the core characteristics of the included studies, while Multimedia Appendix 3 provides the full data extraction framework, developed in accordance with the Cochrane Handbook and PRISMA 2020 recommendations [27,36]. This detailed extraction table promotes transparency, enables systematic comparison across heterogeneous study designs, and facilitates editorial and peer review, although not all extracted items were directly used in the synthesis.

**Table 2.** Summary of basic characteristics of the included studies on the receipt of medicines information (MI) among heterogeneous medicine user groups (n=14).

Authors, years, and country	Aim of the study	Setting	Design	Population	MMAT^a^ score
Surveys, national (n=4)					
Mononen et al [37], 2019, Finland	To explore long-term trends in the receipt of MI^b^ in the general population.	National	Cross-sectional repeated postal survey	Medicine users (n=8822), outpatients, and females (63%‐64%).	5
Hämeen-Anttila et al [38], 2018, Finland	To explore the receipt of MI from the internet among frequent internet users.	National	Cross-sectional online survey	Prescription and OTC^c^ medicine users (n=2489), outpatients, and females (85%).	4
O’Donovan et al [39], 2018, United Kingdom	To explore the receipt of MI on ADRs^d^ in the general population.	National	Cross-sectional on-site survey	Prescription medicine users (n=230), outpatients, and females (61%).	3
Santos et al [40], 2022, Switzerland	To explore the perceptions of conflicting information on chronic medications in the general population.	National	Cross-sectional online or on-site survey	Prescription medicine users (n=405), inpatients and outpatients, and females (57%).	3
Surveys, local (n=6)					
Krska and Morecroft [41], 2013, United Kingdom	To explore the experiences of receiving MI among hospital patients.	Hospitals (n=6) in North West England	Cross-sectional on-site survey	Hospital patients using prescription medicines (n=1218), inpatients, and females (51%).	4
Krska and Morecroft [42], 2013, United Kingdom	To assess the receipt of side-effect information in relation to suspected ADRs among hospital patients.	Hospitals (n=6) in North West England	Cross-sectional on-site survey	Hospital patients using prescription medicines (n=1218), inpatients, and females (51%).	4
Cooper and Garrett [43], 2014, Australia	To explore the experiences and preferences of receiving MI among hospital patients.	Hospitals (n=2) in New South Wales	Cross-sectional on-site survey	Hospital patients using prescription medicines (n=292), hospital-discharged patients, and females (61%).	3
DeLorme et al [44], 2011, United States	To explore the receipt of MI on prescription medicines and source selection among adult medicine users.	Southeastern metropolitan area	Cross-sectional computer-assisted telephone survey	Prescription medicine users (n=234), outpatients, and females (71%).	2
Perry et al [45], 2020, Australia	To explore the receipt of MI and supply behaviors among elite and developing athletes.	State-based sporting institute in Perth	Cross-sectional online survey	Athletes using prescription or nonprescription medicines (n=90) and outpatients.	2
Bergmo et al [46], 2025, Norway	To explore the receipt of MI from the internet among pharmacy customers.	Community pharmacies (n=11) in Tromsø and social media	Cross-sectional online and paper-based survey	Prescription and OTC medicines users (n=303), outpatients, and females (65%).	1
Qualitative interviews (n=2)					
Haverhals et al [47], 2011, United States	To explore medication management processes among adults with multimorbidity.	Metropolitan areas in Denver and Boulder, Colorado	Semistructured individual and group interviews	Prescription medicine users (n=32), outpatients, and females (10%).	5
Tong et al [48], 2018, Australia and the United Kingdom	To explore the receipt of spoken MI and WMI^e^ among adult medicine users.	Australian and United Kingdom consumers	Semistructured individual interviews	OTC medicine users (n=37 in Australia and n=39 in the United Kingdom), outpatients, and females (50%).	5
Mixed methods (n=2)					
Mackridge et al [49], 2018, United Kingdom	To assess the receipt of information about medication changes during admission and post-discharge support among hospital patients.	Hospitals (n=6) in North West England	Study 1: cross-sectional face-to-face survey and study 2: telephone follow-up survey.	Hospital patients using prescription medicines (study 1: n=444 and study 2: n=99), hospital-discharged patients, and females (53% in study 1 and 45% in study 2).	4
Bastholm-Rahmner et al [50], 2018, Sweden	To assess the receipt of MI and knowledge of the list of national recommended essential medicines (Wise List) among hospital patients.	Primary health care centers (n=4) in Stockholm	Study 1: on-site survey (included) and study 2: focus group discussions (not included).	Medicine users (study 1: n=312), outpatients, and females (59%).	3

a MMAT: Mixed Methods Appraisal Tool.

b MI: medicines information.

c OTC: over-the-counter.

d ADR: adverse drug reaction.

e WMI: written medicines information.

**Table 3.** Summary of basic characteristics of the included studies on the receipt of medicines information (MI) among adults using specific medicines (n=12).

Authors, year, and country	Aim of the study	Setting	Design	Population	MMAT^a^ score
Surveys, national (n=3)					
Amundsen et al [51], 2016, Norway	To explore the use of antimigraine medications and information needs during pregnancy and breastfeeding.	National	Cross-sectional online survey	Pregnant and breastfeeding women using antimigraine medicines (n=401), outpatients, and females (100%)	5
Leonardo et al [52], 2020, Australia	To explore the receipt of MI^b^ on methotrexate treatment among patients with rheumatoid arthritis.	National	Cross-sectional online survey	Patients with rheumatoid arthritis using or having used methotrexate (n=742), outpatients, and females (76%)	5
Otón et al [53], 2023, Spain	To assess the information needs and expectations in clinical care among methotrexate users.	National	Cross-sectional online survey	Patients with immune-mediated disease using methotrexate (n=283), outpatients, and females (82%)	2
Surveys, local (n=6)					
Bults et al [54], 2012, The Netherlands	To explore the receipt of MI on Q fever vaccination among patients with cardiovascular conditions.	Municipal public health service	Questionnaire-based individual interviews	Cardiovascular medicine users (n=413), outpatients, and females (39%)	5
Houser et al [55], 2016, United States	To assess sociodemographic differences in the receipt of prescription NSAID^c^ risk information among adult medicine users.	Physician practices (n=39) in Alabama	Cross-sectional computer-assisted telephone survey	Prescription NSAID or OTC^d^ medicine (ibuprofen or naproxen) users (n=220), outpatients, and females (75%)	5
de Toro et al [56], 2019, Spain	To assess the receipt of MI about biological medicines among patients with arthritis.	Hospitals (n=50)	Cross-sectional on-site survey	Arthritis medicine (s.c. biological) users (n=592), outpatients, and females (58%)	4
Geryk et al [57], 2016, United States	To explore the receipt of MI among patients with arthritis.	Participants were recruited in several ways	Cross-sectional online survey	Arthritis medicine users (n=328), outpatients, and females (79%)	4
Carpenter et al [58], 2011, United States	To explore the receipt of MI and the perceived credibility of MI sources among patients with vasculitis.	Participants were recruited in several ways	Cross-sectional online survey	Vasculitis medicine users (n=232), outpatients, and females (69%)	3
Carpenter et al [59], 2014, United States	To explore the sources of conflicting MI among patients with arthritis.	Participants were recruited in several ways	Cross-sectional online survey	Arthritis medicine users (n=328), outpatients, and females (79%)	3
Qualitative interviews (n=3)					
Bergsholm et al [60], 2023, Norway	To explore the receipt of MI among antibiotic users.	Central urban areas in the Norwegian South-Eastern health region	Focus group interviews (n=2)	Antibiotic medicine users (n=8), outpatients, and gender not reported	5
Hayden et al [61], 2015, United Kingdom	To explore the receipt of MI and adherence-related beliefs about methotrexate among patients with arthritis.	University Hospitals of Leicester NHS^e^ Trust	Semistructured individual interviews	Patients with arthritis using oral methotrexate (n=15), outpatients, and females (73%)	5
Wakob et al [62], 2022, Germany	To assess the benefits and risks of medication and the receipt of MI among patients with cardiovascular conditions.	University Hospital (n=1) offering tertiary care	Semistructured individual interviews	Cardiovascular medicine users (n=102), inpatients, and females (35%)	5

a MMAT: Mixed Methods Appraisal Tool.

b MI: medicines information.

c NSAID: nonsteroidal anti-inflammatory drug.

d OTC: over-the-counter

e NHS: National Health Service.

### Data Synthesis and Analysis

Before selecting the analytical approach, the feasibility of conducting a meta-analysis was carefully evaluated in accordance with established methodological guidance on evidence synthesis [63]. Quantitative pooling was deemed inappropriate because the included studies were not sufficiently comparable to support a meaningful pooled estimate. The studies differed substantially in their target populations, settings (eg, hospitals, outpatient care, and community pharmacies), types of medicines, and in how the receipt of MI and MI sources were defined and operationalized. Outcome measures also varied considerably across studies.

Several methodological limitations further reduced confidence in the robustness and interpretability of any potential summary effect. These included low or unreported response rates, the use of convenience samples or online surveys with uncertain denominators, and variable study quality across designs. Pooling effect estimates from such studies would risk producing a summary effect with questionable validity and misleading precision. Moreover, although meta-analysis can be used to explore heterogeneity, the limited number of studies within meaningful subgroups, combined with diverse outcome measures and variable study quality, would make any statistical exploration of heterogeneity unreliable and potentially misleading. Taken together, these factors indicated that quantitative synthesis would not yield a valid or interpretable summary estimate. No sensitivity analyses were conducted due to the absence of quantitative pooling.

Consequently, a narrative synthesis was conducted as the primary analytic approach, allowing transparent interpretation of patterns and trends in the evidence without implying a summary effect that could not be meaningfully estimated. Given the heterogeneity of study designs, outcome measures, and the operationalization of MI-related constructs, the synthesis followed a structured qualitative analytic process. In this process, the MI sources reported in each study were examined, definitions and distinctions of internet-based MI sources were reviewed, and any theoretical frameworks were identified.

In qualitative studies, formal coding procedures were not used. Instead, key findings relevant to the research objectives were systematically identified and summarized. This approach enabled qualitative evidence to be integrated into the narrative synthesis in a manner comparable to the treatment of quantitative findings, while maintaining transparency regarding how qualitative insights informed the overall conclusions.

To enhance analytical clarity, the studies were further classified into two main categories: (1) studies examining the receipt of MI among heterogeneous adult medicine user groups and (2) studies examining the receipt of MI among adults using specific medicines. Results were reported within these categories. Finally, a summary table and a visual representation of the key findings were developed to provide a comprehensive overview of the available evidence.

### Ethical Considerations

No ethics board review was required for this study, as it involved the analysis of publicly available, anonymized data only, in accordance with the Finnish National Board on Research Integrity (TENK, 2019).

## Results

### Characteristics of Included Studies

**Figure 1.**
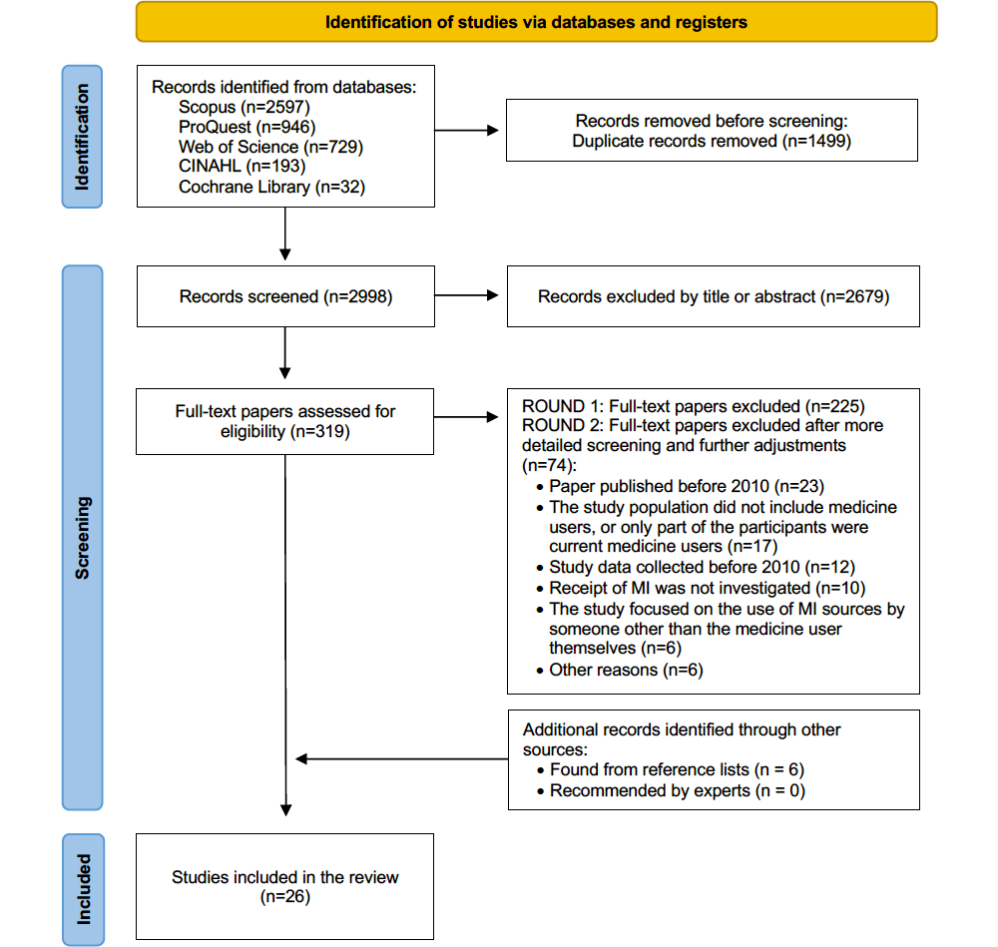
PRISMA (Preferred Reporting Items for Systematic Reviews and Meta-Analyses) 2020 flow diagram for the literature screening covering the time period 2010‐2025. MI: medicines information.

A total of 26 studies met the inclusion criteria (Figure 1) [37-62]. An overview of the core characteristics of the included studies is presented in Table 2, while the full data extraction, including detailed methodological and contextual information, is provided in Multimedia Appendix 3, representing 19,891 medicine users from 10 different countries. No systematic reviews or meta-analyses were identified among the included studies.

More than half of the studies (n=16, 62%) were carried out in European countries (Tables2  4) [37-42,46,49-51,53,54,56,60-62]. Six (23%) studies were conducted in the United States [44,47,55,57-59] and 3 (12%) in Australia [43,45,52]. One study used a multinational design and was conducted in both Australia and the United Kingdom [48]. The predominant study designs were cross-sectional surveys (n=19, 73%), performed by mail, online, telephone, or questionnaire-based interviews [37-46,51,53-59,62] and qualitative interviews (n=5, 19%) [47,48,60-62]. Most studies were conducted among outpatients (n=20, 77%) [37-39,44-48,50-61], while 3 studies focused on inpatients [41,42,62]. Two studies examined patients at the time of hospital discharge [43,49], and 1 study included both inpatients and outpatients [40]. Approximately half of the studies (n=14, 54%) investigated heterogeneous medicine user groups [37-50], while the remaining studies (n=12, 46%) focused on specific medicine user groups [51-62]. Across the included studies, the most commonly examined MI sources were HCPs (eg, physicians, pharmacists, nurses, and midwives), the internet and social media, PLs, and family and friends (Table 2 and Multimedia Appendix 3).

**Table 4.** Characteristics of the included studies (n=26).

Category and subcategory	Frequency
Study country	
United States [44,47,55,57-59]	6
United Kingdom [39,41,42,49,61]	5
Australia [43,45,52]	3
Norway [46,51,60]	3
Finland [37,38]	2
Spain [53,56]	2
Australia and the United Kingdom [48]	1
Germany [62]	1
The Netherlands [54]	1
Sweden [50]	1
Switzerland [40]	1
Study design	
Survey, local, and cross-sectional [41-46,54-59]	12
Survey, national, and cross-sectional [37-40,51-53]	7
Qualitative interview [47,48,60-62]	5
Mixed methods [49,50]	2
Study population	
Heterogeneous medicine user groups	14
Prescription medicine users [39,40,42,44,47,49]	6
Not specified [37,38,41,43,50]	5
Prescription and OTC^a^ medicine users [45,46]	2
OTC medicine users [48]	1
Specific medicine user groups	12
Arthritis medicine users [55-57,59]	4
Methotrexate users [52,53,61]	3
Cardiovascular medicine users [54,62]	2
Antibiotic medicine users [60]	1
Antimigraine medicine users [51]	1
Vasculitis medicine users [58]	1
Study context	
Outpatients [37-39,44-48,50-61]	20
Inpatients [41,42,62]	3
Hospital-discharged patients [43,49]	2
Inpatients and outpatients [40]	1
MI^b^ sources studied	
Physician and specialist [37-41,44-47,49-62]	23
Pharmacist [37-41,43-49,51,52,55-60,62]	21
Internet and social media [37,38,40,42,44-47,50-52,55,57-62]	18
Package leaflet (PL) [37-39,41,42,44,46-49,51,53,57-59,61,62]	17
Family^c^, relatives, and friends [37,42,44,46,47,52-59,62]	14
Nurse and midwife [37-39,41,47,49,51,55-58]	12
Books, magazines, and newspapers [37,42,44,57-59]	6
Radio, television, and media [37,44,56,59,62]	5
Advertisements [37,46,57,59]	4
Health care professional (HCP), not specified [42,49,55,59]	4
Patient brochures and pamphlets [56-59]	4
Written medicines information (WMI), not specified [48,53,55,60]	4
Newsletters [57-59]	3
Support groups [57-59]	3
Medical books, drug handbooks, and health journals [44,47]	2
Podcasts [57,59]	2
WMI from HCPs [41,44]	2
Health food stores [37]	1
Hospitals [45]	1
Other patients [56]	1
Patient associations [56]	1
Pharmaceutical companies [44]	1
Telephone services and MI centers [37]	1

a OTC: over-the-counter.

b MI: medicines information.

c Including spouse and partner.

### Methodological Quality

Among the included studies (n=26), 10 (38%) studies met all MMAT methodological quality criteria (5 points) (Table 5 and Multimedia Appendix 3) [37,47,48,51,52,54,55,60-62]. Six (23%) studies fulfilled 4 of the 5 criteria (4 points) [38,41,42,56-58], 6 (23%) studies met 3 criteria (3 points) [39,40,43,49,50,59], 3 (12%) studies met 2 criteria (2 points) [44,45,53], and 1 (4%) study met 1 criterion (1 point) [46] (Multimedia Appendix 1 and Table 4). All qualitative (n=5) studies fulfilled all MMAT criteria (5 points) [47,48,60-62]. The 2 mixed methods studies fulfilled 3-4 criteria (3‐4 points) [49,50]. Among quantitative (n=19) studies, 5 met all criteria (5 points) [37,51,52,54,55], 5 fulfilled 4 criteria (4 points) [38,41,42,56,57], 5 fulfilled 3 criteria (3 points) [39,40,43,58,59,62], and 3 fulfilled 2 criteria (2 points) [44,45,53]. One quantitative study fulfilled only 1 criterion (1 point) [46].

The methodological characteristics that best met the quality criteria in quantitative studies (n=19) were the following: statistical analysis was appropriate to answer the research questions (18 studies met the criteria) [37-45,47,51-59]; the measurements were appropriate (18 studies) [37-46,51-55,57-59]; and a relevant sampling strategy to address the research question was applied (14 studies) (Table 5) [37,38,40-43,51,52,54-59]. The most common limitation in quantitative studies was that the risk of nonresponse bias was either rated as low without sufficient justification (9 studies) [41,42,45,49,50,53,57-59] or was not reported at all (6 studies) [38-40,43,44,46]. Only 3 studies incorporated theoretical frameworks: (1) Protection Motivation Theory and the Health Belief Model [54], (2) Comprehensive Model of Information Seeking [44], and (3) Necessity-Concerns Framework and Cognitive Dissonance Theory [61].

The heterogeneity observed across study designs, populations, outcomes, and analytical approaches precluded quantitative synthesis. Consequently, a qualitative descriptive analysis was conducted.

**Table 5.** Methodological quality assessment of the included studies (n=26) using the Mixed Methods Appraisal Tool (MMAT), organized according to MMAT quality score. Randomized controlled trials (methodological group 2) and nonrandomized studies (methodological group 3) are not presented, as none of the included studies used these study designs.

Study	Screening questions		Subquestions															MMAT^b^ score^a^
			Qualitative studies					Quantitative descriptive studies					Mixed method studies					
	S1	S2	1.1	1.2	1.3	1.4	1.5	4.1	4.2	4.3	4.4	4.5	5.1	5.2	5.3	5.4	5.5	
Amundsen et al [51]	Yes	Yes						Yes	Yes	Yes	Yes	Yes						5
Bergsholm et al [60]	Yes	Yes	Yes	Yes	Yes	Yes	Yes											5
Bults et al [54]	Yes	Yes						Yes	Yes	Yes	Yes	Yes						5
Haverhals et al [47]	Yes	Yes	Yes	Yes	Yes	Yes	Yes											5
Hayden et al [61]	Yes	Yes	Yes	Yes	Yes	Yes	Yes											5
Houser et al [55]	Yes	Yes						Yes	Yes	Yes	Yes	Yes						5
Leonardo et al [52]	Yes	Yes						Yes	Yes	Yes	Yes	Yes						5
Mononen et al [37]	Yes	Yes						Yes	Yes	Yes	Yes	Yes						5
Tong et al [48]	Yes	Yes	Yes	Yes	Yes	Yes	Yes											5
Wakob et al [62]	Yes	Yes	Yes	Yes	Yes	Yes	Yes											5
de Toro et al [56]	Yes	Yes						Yes	Yes	No	Yes	Yes						4
Geryk et al [57]	Yes	Yes						Yes	Yes	Yes	Cannot tell	Yes						4
Hämeen-Anttila et al [38]	Yes	Yes						Yes	Yes	Yes	No	Yes						4
Krska and Morecroft [41]	Yes	Yes						Yes	Yes	Yes	Cannot tell	Yes						4
Krska and Morecroft [42]	Yes	Yes						Yes	Yes	Yes	Cannot tell	Yes						4
Mackridge et al [49]	Yes	Yes						Yes	Yes	Yes	Cannot tell	Yes						4
Bastholm-Rahmner et al [50]	Yes	Yes	Yes	Yes	Yes	Yes	Yes	Yes	No	Yes	Cannot tell	Yes	Yes	Yes	Yes	Yes	No	3
Carpenter et al [58]	Yes	Yes						Yes	No	Yes	Cannot tell	Yes						3
Carpenter et al [59]	Yes	Yes						Yes	No	Yes	Cannot tell	Yes						3
Cooper and Garrett [43]	Yes	Yes						Yes	No	Yes	No	Yes						3
O´Donovan et al [39]	Yes	Yes						No	Yes	Yes	No	Yes						3
Santos et al [40]	Yes	Yes						Yes	No	Yes	No	Yes						3
DeLorme et al [44]	Yes	Yes						No	No	Yes	No	Yes						2
Otón et al [53]	Yes	Yes						No	No	Yes	Cannot tell	Yes						2
Perry et al [45]	Yes	Yes						No	No	Yes	Cannot tell	Yes						2
Bergmo et al [46]	Yes	Yes						No	No	Yes	No	No						1

a MMAT score: 5=100% compliance with all quality evaluation criteria (high confidence), 4=80% of quality evaluation criteria met (moderate confidence), 3=60% of quality evaluation criteria met (low confidence), 2=40% of quality evaluation criteria met (very low confidence), 1=20% of quality evaluation criteria met, and 0=0% of quality evaluation criteria met.

b MMAT: Mixed Methods Appraisal Tool.

### Receipt of MI Among Heterogeneous Medicine User Groups

A total of 14 studies examined the receipt of MI among heterogeneous medicine user groups (Table 2 and Multimedia Appendix 3) [37-50]. Of these, 8 studies focused on users of unspecified prescription medicines [39-44,47,49], 3 on users of both prescription and over-the-counter (OTC) medicines [38,45,46], 2 on general medicine users [37,50], and 1 on OTC medicine users exclusively [48]. The studies were performed in the United Kingdom [39,41,42,49], Australia [43,45], Finland [37,38], Norway [46], Switzerland [40], the United States [44,47], and Sweden [50], with 1 multinational study carried out in Australia and the United Kingdom [48]. Most studies were conducted in the early 2010s, as 7 studies between 2010 and 2016 [37,38,41,42,48-50], 3 between 2017 and 2025 [40,45,46], while 4 studies did not report the year of data collection [39,43,44,47]. Cross-sectional surveys were the most frequently used study design (n=10) [37-46]. Qualitative interviews were used in 2 studies [44,49], and mixed methods were applied in 2 studies [49,50]. Most studies were conducted among outpatients (n=9) [37-39,44-48,50], while 2 studies focused on hospital inpatients [41,42], 2 on hospital-discharged patients [43,49], and 1 included both inpatients and outpatients [40].

**Figure 2.**
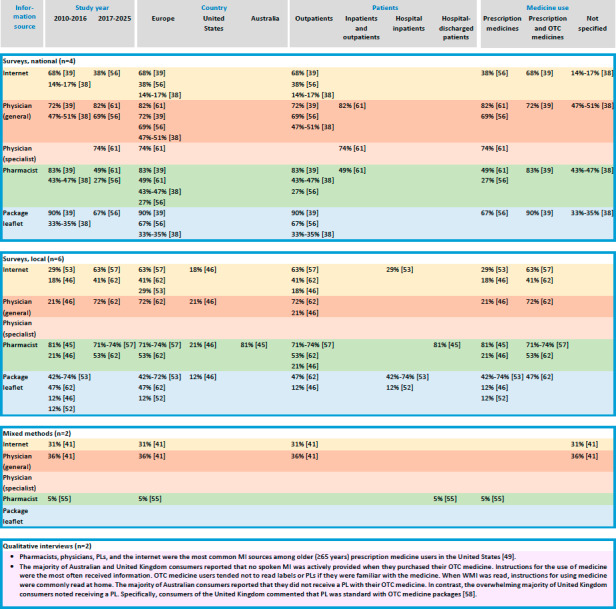
Summary of findings from the studies (n=14) on the receipt of medicines information (MI) via the internet and commonly used information sources among heterogeneous medicine user groups in 2010‐2025. The figure contains only the results reported in the included studies. OTC: over-the-counter; PL: package leaflet; WMI: written medicines information.

Across the heterogeneous medicine user (n=14) studies, a consistent finding was that physicians, community pharmacists, and PLs were the most commonly reported MI sources, regardless of study design, study period, population, or country (Figure 2 and Multimedia Appendix 3) [37-50]. However, notable differences were observed between prescription and OTC medicine users. Physicians, pharmacists, and PLs were the primary information sources related to prescription medicines [39,40,42-47,49]. In contrast, pharmacists were the most common information sources for OTC medicines, particularly in situations involving symptom-based requests, first-time purchases, or when consumers actively sought advice from pharmacists [48]. Nevertheless, 38% of medicine users reported not receiving any MI from HCPs regarding their medicines [37].

The receipt of MI varied substantially by research method, patient group, and type of medicine use (Figure 2 and Multimedia Appendix 3). No clear temporal trends in the receipt of MI were identified. National and local online surveys conducted in Europe indicated that 63%‐68% of prescription and OTC medicine users received MI from the internet [38,45]. In contrast, substantially lower proportions (14%‐41%) were reported in studies using other research methods, such as paper-based surveys, computer-assisted surveys, and focus group discussions conducted in Europe and North America (ie, the United States) [37,39,42,44,46,50]. According to national online and on-site surveys from Europe, 69%‐72% of adult prescription and OTC medicine users received MI from general practitioners [38,39]. Lower proportions (21%‐51%) were reported in national postal and local computer-assisted surveys conducted in Europe and North America [37,44]. Pharmacists were reported as MI sources by 81% of hospital-discharged patients in Australia based on an on-site survey [43]. Among European outpatients, 71%‐83% received MI from pharmacists according to national and local online surveys [38,45], whereas lower proportions (27%‐49%) were reported in national surveys using other data collection methods [37,39,40]. In North America, a local computer-assisted survey indicated that 21% of outpatients received MI from pharmacists [44]. Although the PLs are among the most commonly reported MI sources, not all medicine users read them [37-39,41,42,44]. In a national online survey from Finland, 90% of prescription and OTC medicine users reported reading PLs [38]. In comparison, 12%‐67% of medicine users reported reading PLs in national and local surveys using nononline methods in Europe and North America [37,39,41,44]. Among hospital patients in the United Kingdom, 74% reported usually reading PLs, whereas 19% never read them, and 7% read them only when unexpected adverse drug reactions occurred [42].

Qualitative interviews from the United States indicated that older medicine users (≥65 years) most commonly received MI from pharmacists and physicians, followed by PLs and the internet (Figure 2 and Multimedia Appendix 3) [47]. Qualitative interviews from Australia and the United Kingdom further suggested that consumers often did not receive active spoken MI when purchasing OTC medicines [48]. OTC medicine users tended not to read labels or PLs when they were familiar with the product, and when WMI was read, it was commonly read at home rather than at the point of purchase. In the Australian data, many respondents reported not receiving a PL with their OTC medicines [48]. In contrast, respondents from the United Kingdom more frequently reported receiving PLs, with some noting that PLs were typically included in OTC medicine packages [47,48].

### Receipt of MI Among Specific Medicine User Groups

Altogether, 12 studies examined the receipt of MI among adults using specific medicines for various conditions, including arthritis [55-57,59], methotrexate therapy [52,53,61], cardiovascular medications [54,62], antibiotic medications [60], antimigraine treatments [51], and vasculitis medications [58] (Table 2, Multimedia Appendix 3). The majority of the studies were carried out in the early 2010s, with 7 studies performed between 2010 and 2016 [51,54-57,59,61], 4 studies between 2017 and 2025 [52,53,60,62], and 1 study in which the year of data collection was not reported [58]. The studies were conducted in several countries, including the United States [55,57-59], Spain [53,56], Germany [62], the Netherlands [54], Norway [60], and Australia [52]. A range of research methods was used, with cross-sectional surveys being the most common design (n=8) [51-53,55-59]. Of these, 3 studies used national online surveys [51-53]. In addition, 3 studies used qualitative interviews [60-62], and 1 study used questionnaire-based interviews [54]. Most studies (n=11) were conducted among outpatients [51-61], while 1 study focused on hospital inpatients [62].

**Figure 3.**
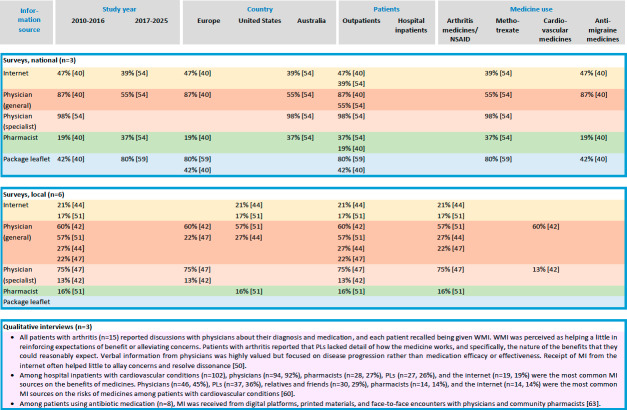
Summary of findings from the studies (n=12) on the receipt of medicines information (MI) via the internet and commonly used information sources among specific medicine user groups in 2010‐2025. The figure contains only the results reported in the included studies. PL: package leaflet; WMI: written medicines information.

Consistent with findings from studies involving heterogeneous medicine user groups, physicians, pharmacists, and PLs were the most frequently reported MI sources across specific medicine user groups, irrespective of study design, study period, or country in which the research was conducted (Figure 3 and Multimedia Appendix 3) [51-62]. No clear temporal trends in the receipt of MI were observed over time. However, the frequency of MI receipt varied by research method, patient group, and type of medicine. For instance, among Australian outpatients using methotrexate (n=742), specialist physicians (98%), PLs (80%), and general physicians (55%) were the most commonly reported MI sources in a national online survey [52]. In contrast, a national online survey of pregnant and breastfeeding women using antimigraine medicines (n=294) identified general physicians (87%) as the primary MI source, followed by the internet (47%) and PLs (42%) [51]. In local surveys, general physicians were the leading MI sources for cardiovascular medicine users in the Netherlands (60%, n=413) [54], whereas specialist physicians (75%) and general physicians (22%‐57%) predominated among arthritis medicine and nonsteroidal anti-inflammatory drug users in Spain and the United States [55,56,59].

In a qualitative interview study from the United Kingdom, arthritis outpatients (n=15) reported discussing their diagnosis and medication with physicians, with each patient recalling receipt of WMI [61]. While WMI provided some reassurance about benefits and alleviated concern, patients felt it lacked detail on how the medicines worked and the specific benefits they could expect. Though highly valued, verbal information from physicians focused more on disease progression than on medication efficacy. MI from the internet rarely helped resolve concerns or clarify discrepancies. Among cardiovascular hospital inpatients in Germany (n=102), physicians (92%), pharmacists (27%), and PLs (26%) were the most common MI sources regarding medication benefits, whereas physicians (45%) and PLs (36%) were the primary information sources about medication risks [62]. A Norwegian qualitative focus group study among patients using antibiotic medication (n=8) reported that patients received MI from 3 main sources: digital platforms, printed materials, and face-to-face encounters with physicians and community pharmacists [60].

### Internet as a Source of MI

Receipt of MI from the internet varied substantially by population, methodology, and country (Figures2  3; Multimedia Appendix 3) [37,38,40,42,44-47,50-52,55,57-62]. Receipt of MI from the internet was more common among women, individuals younger than 65 years, those with polytechnic, college, or university degrees, and frequent internet users [37,38,40,46,58]. Based on the long-term population-based trend survey from Finland [37], the interview study from Germany [62], and the local cross-sectional surveys from Sweden [50], the United Kingdom [42], and the United States [44,55], 14%‐31% of adult medicine users reported receiving MI online. However, online surveys from Australia [45,52], Finland [38], and Norway [51], and a paper- and web-based survey from Norway [46] showed higher usage, with 37%‐68% of medicine users receiving MI from the internet. Certain medicine user groups were more likely to receive MI online; for example, 47% of pregnant and breastfeeding women using antimigraine medicines [51], and 39% of patients with rheumatoid arthritis using or having used methotrexate [52] had received MI from the internet. In comparison, 19% of patients with cardiovascular conditions [62] and 17% of nonsteroidal anti-inflammatory drug users [55] did so.

Medicine users received MI most commonly through health portals, search engines, pharmacy websites, patient organizations’ websites, and medicine authorities’ websites [38,46,47,52,60,61]. Patients with chronic illness often turned to patient organizations’ websites (33%), while patients with mental illness (32%) and patients with thyroid disease (26%) used discussion forums [38]. Younger individuals and those who found advertising-based internet sources useful were more likely to use these platforms [44].

The internet was typically used for quick, supplementary information, often in conjunction with other MI sources [42,47,60]. However, some patients reported feeling overwhelmed by the sheer volume of MI available online [47], with most receiving MI from an average of 1 to 3 different websites [38,46]. While some medicine users were skeptical about the reliability of online sources, others found that receiving MI exacerbated concerns or dissonance [46,47]. The receipt of MI from the internet often did little to help allay concerns and resolve dissonance, but on occasion, it was reported to exacerbate it by raising further concerns [60,61]. However, some patients resorted to avoidance strategies to limit exposure to conflicting MI or disinformation or to refrain from reading official patient information to reduce anxiety about side effects.

### Medicine Users Encountering Conflicting MI

Among adult prescription medicine users, 47% had sometimes or often received conflicting MI regarding their medication, primarily regarding side effects (22%) and duration of treatment (18%) [40]. Conflicting MI was most commonly received from general practitioners (82%), specialists (74%), and pharmacists (49%). Younger medicine users (18‐50 years) were more likely to encounter conflicting MI from the internet and social media than older users (>50 years) [40,59]. Factors associated with more conflicting MI included being prescribed more than 3 medicines, higher perceived disease severity, poor medication adherence, younger age (<50 years), higher education, non-White race, and using more MI sources [40,59]. However, gender, language, nationality, and duration of chronic diseases showed no significant differences in perceptions of conflicting MI [40]. Some medicine user groups have experienced conflicting MI more than others, such as patients with arthritis (80%) [59], pregnant women (45%) [51], and patients with cardiovascular conditions (37%) [40]. In response to conflicting MI, 45% of users sought a follow-up appointment with the same physician, and 30% sought additional medical advice [40]. Furthermore, 34%‐39% of medicine users either discontinued the medication or became nonadherent following conflicting MI [40,51].

### Number of MI Sources Used

Most adult medicine users received MI on their medication from multiple information sources [37,38,44,46,51,52]. Commonly, younger and more educated, as well as those with greater medical–related concerns, tended to receive and seek MI from a greater number of sources than others [46,52]. Typically, MI was received from 2 to 4 sources [37,44,46,51,52]. Internet users, on average, consulted 1-3 different websites for MI [38,46]. On the other hand, a population-based survey indicated that 28% of adult medicine users reported not receiving information about their medication from any source [37].

## Discussion

### Principal Findings

Despite increasing attention to the quality and accessibility of MI to medicine users in recent decades, both nationally and globally, only a limited number of studies have focused on the receipt of MI among adult medicine users over the past 15 years (2010‐2025). Concurrently, the variety of MI sources available to consumers and medicine users has expanded considerably, particularly with the growth and diversification of electronic information channels. Nearly all studies included physicians, pharmacists, PLs, and the internet as MI sources, with considerable variation in other sources across studies. Only 1 national long-term trend study was identified [2,37], while most studies were descriptive cross-sectional designs, and only 3 applied theory-driven measures [44,54,61].

The limited and heterogeneous use of theoretical frameworks highlights a persistent gap in the current MI research. Of the included studies, three explicitly applied theoretical approaches: (1) “Protection Motivation Theory and the Health Belief Model” [54], (2) the “Comprehensive Model of Information Seeking” [44], and (3) the “Necessity-Concerns Framework” combined with “cognitive dissonance theory” [61]. Although these frameworks provide valuable insights into health behavior, information seeking, and medication-related beliefs, they were developed more than a decade ago and predate many conceptual advances that have since shaped contemporary health and digital communication research. They were formulated before the widespread adoption of social media, mobile health technologies, and algorithmically curated content, and therefore may not fully capture emerging phenomena, such as digital trust, online credibility assessment, and the interactive nature of current MI environments.

Recent theoretical developments, such as updated technology acceptance models, empowerment-oriented frameworks, and expanded conceptualizations of health literacy, medication literacy, communication processes, and adherence models, offer more comprehensive tools for understanding how adults seek, receive, interpret, evaluate, and act upon MI in increasingly digitalized contexts. Integration of these frameworks can enhance methodological rigor by guiding study design, clarifying key constructs, and supporting consistent interpretation of findings across heterogeneous studies.

The growing role of AI-based tools further underscores the need for theoretically grounded research. AI-driven platforms introduce new dimensions related to algorithmic mediation, personalization, user-system interaction, and evolving trust dynamics, effectively repositioning MI sources within a rapidly transforming digital ecosystem [64]. Future MI research would benefit from more explicit theory-driven approaches and conceptual models that reflect contemporary digital health communication processes and the increasing influence of AI-based information systems.

The majority of studies originated from Europe (17/26 studies), followed by the United States (6/26) and Australia (4/26). In recent decades, the EU Pharmaceutical Forum, established in 2002, has played a key role in shaping and coordinating MI practices across Europe [2,31,32]. This high-level forum aimed to develop alternative strategies for enhancing consumer access to quality MI, particularly after the EU‘s ban on direct-to-consumer advertising of prescription medicines. The forum’s recommendations were published in 2008, with special emphasis on national coordination of MI, increasing the availability and use of electronic MI, and enhancing cooperation between public and private MI providers [32]. This framework has likely influenced research activities and the implementation of strategies for reliable MI throughout the EU [1,2], as can be seen in our systematic review. For instance, Finland established its ongoing National MI Strategy in 2012, inspired by the EU Pharmaceutical Forum’s recommendations [1,2,65-68].

The introduction of statutory PLs marked a significant milestone in the EU’s MI policies in the late 1990s [11,69]. Europe remains the only continent where PLs are mandatory on all pharmaceutical packaging [70]. The implementation of PLs was planned in the United States as early as the 1970s, but the mandate was only partially realized [70]. Although PLs may not encompass the full spectrum of MI in terms of structure and content [71-77], they provide an essential, easily accessible source of basic, product-specific information for medicine users. The importance of PLs is also evidenced by our systematic review, which found that PLs were among the top 3 MI sources in all the included studies, regardless of the research method, study year, population, or country of origin. Since the 1970s, the active development of PLs and related research has been particularly prominent within the EU, leading to the establishment of EU-approved readability guidelines and mandatory user testing for PLs [11,12,70-73,78]. Today, in addition to the PLs inserted in medicinal packages, PLs are made available electronically, in spoken formats, and in Braille, further enhancing access to essential MI for medicine users with special needs [11,69].

Finland stands out as the only country where we found long-term, population-level research on consumers’ and medicine users’ receipt of MI, covering the period from 1999 to 2014 [37]. Initially, this trend study was conducted annually by the National Public Health Institute as part of the national health behavior postal survey, which represented the adult population aged 15‐64 years (conducted during 1978‐2014). In 2015, the Finnish Medicines Agency established a new national biennial population survey, the Medicines Barometer, focusing on timely medicine-related topics important for planning national policies and strategies promoting rational pharmacotherapy. The Medicines Barometer has included a question concerning the receipt of MI among adult Finns in 2017 and 2023 [79,80]. Since 2019, this barometer survey has been conducted exclusively as an online survey. The question about the receipt of MI has been repeated in a comparable format since 1999. Such national trend studies would also be valuable in other countries, as they provide essential data to inform national decision-making in health policy and planning. A similar national trend study on the receipt of WMI was used in the United States by the Food and Drug Administration (FDA) during the 1970s to improve consumers’ access to MI [81]. Another period of national follow-up studies on the receipt of MI in the United States was conducted in the 1990s [77].

The internet as an MI source has been mainly studied at the general level, with various studies examining its role and prevalence of use across different contexts. The internet as an MI source has rarely been studied in its subtypes, such as open-source and open-access MI databases, social media–sharing networks, community forums, blogs, consumer review networks, and different apps. The existing studies have mainly used “rough” measures, overlooking the diverse range of health- and MI-related content available online. A notable trend has been the integration of health and MI into health portals and other electronic sources, including those provided by authorities or commercial entities such as the pharmaceutical industry [2].

This systematic review indicates that although the internet has primarily been studied as a general electronic source of MI, the ongoing digitalization and proliferation of electronic information channels underscore the need to investigate other specific digital MI sources and care pathways. These may include official regulatory websites, online discussion forums, and real-time chat platforms. Such sources should be examined as distinct categories in research, rather than being aggregated under the general label of “internet as a source of MI.” It is crucial to determine where users predominantly access MI online and which websites they consider reliable and credible for health and MI. Future research should also increasingly address the role and impact of misinformation and disinformation on medicine users’ perceptions of health, decision-making processes, and health-related behaviors in the digital environment [17]. These factors may have growing implications for treatment outcomes and the effective implementation of therapeutic interventions.

Our findings indicate that the receipt of MI among adult medicine users has diversified during the 2010s. Medicine users increasingly receive MI from multiple sources, as the internet makes it easy and fast to access various types of information. They also seem to commonly receive MI from traditional sources, such as HCPs (eg, physicians, pharmacists, and nurses), media (eg, television, radio, and newspapers), the internet, specific patient-directed information sources (eg, PLs and telephone services), and personal contacts (eg, family, friends, and peers). Future research should focus on investigating the extent, reliability, and impact of commercial information as an MI source, use more theory-driven study designs and measures to gain a more comprehensive understanding of the receipt of MI among medicine users, and address the need for the development of existing MI sources from the medicine user perspective. It is also important to plan more strategic MI research so that it can cover patients and medicine users from a much larger range of conditions, including self-medication, than is the case in current research.

### Strengths and Limitations

This systematic review provides a comprehensive overview of the receipt of MI among adult medicine users across diverse settings in developed countries. A further strength is the systematic examination of how MI sources were defined and operationalized across studies, enabling transparent comparison of patterns despite differences in study designs.

A key finding was the substantial heterogeneity across the included studies, spanning study designs, populations, settings, operationalizations of MI, and analytical approaches. As detailed in Multimedia Appendix 3, studies varied markedly in recruitment strategies, definitions and measurement of MI sources, and outcome measures. This multidimensional heterogeneity, particularly in methodology, population, medication in use, and MI sources, precluded conducting a meta-analysis and supports the use of a narrative synthesis to interpret patterns in the receipt of MI. Importantly, pooling effect estimates from studies with these limitations would have risked producing a summary effect with questionable validity and misleading precision. In addition, although meta-analysis can be used to explore heterogeneity, the limited number of studies within meaningful subgroups would have made such analyses difficult. Combined with diverse outcome measures and variable study quality, any statistical exploration of heterogeneity would have been unreliable and potentially misleading.

The restriction to English-language, peer-reviewed full-text papers may have introduced both language and publication bias. In addition, differences in health care systems and service delivery contexts (eg, hospital, outpatient care, and community pharmacy) likely influenced access to and use of MI sources, limiting cross-study comparability. The studies were conducted in diverse settings, which may have substantially impacted the results. Nearly half of the included studies (17/26, 65%) originated from a variety of European countries [37-42,46,49-51,53,54,56,60-62], which may affect generalizability to other regions.

Methodological limitations in the quantitative studies further reduced confidence in the evidence base. Notably, nonresponse bias was often insufficiently assessed or not reported, and was sometimes rated as low or reported without adequate justification despite low or uncountable response rates (eg, convenience samples and online surveys), reducing confidence in the robustness and generalizability of the evidence. Given the broad scope of the topic, it was necessary to refine and expand the predetermined exclusion criteria during the screening process. Applying these stringent criteria ensured that the review remained focused on the receipt of MI among adult medicine users. However, this inevitably resulted in the exclusion of a substantial number of studies not directly aligned with the objective of this review.

Although a range of research methods was used, the majority of studies were cross-sectional, using a single cross-sectional design (18/26, 69%) [38-46,51-53,55-60], and many relied on online surveys, potentially overrepresenting respondents who are more easily reached through digital platforms. Only 1 study analyzed long-term trends in the receipt of MI more than 16 years (from 1999 to 2014) [37], highlighting the critical need for further long-term, population-based analyses using diverse MI sources among medicine users. It is essential to recognize that none of the reviewed studies used a longitudinal design. The studies also did not separate internet sources into different subcategories, limiting the ability to assess the internet as a multidimensional MI source. Furthermore, only 3 studies used a theoretical framework [44,54,61], highlighting significant gaps to be addressed in future research.

A major limitation of the reviewed literature is the lack of population-level studies assessing the receipt of internet-based MI. Existing national studies from Finland indicate that the prevalence estimates are substantially lower in representative population samples than in online surveys or selected specific medicine user groups [2,37]. This pattern is corroborated by recent national monitoring studies in Finland, which show that, at the population level, the internet has not yet reached the same prevalence as traditional MI sources (eg, physicians, community pharmacists, and PLs) [79,80].

Because the review intentionally included only studies from developed countries, the findings predominantly apply to this context and do not reflect the situation in developing countries. Consequently, generalization should consider differences in MI practices, including accessibility and use of WMI and internet sources across countries and continents. Future research should investigate the receipt of MI in middle- and low-income countries, as well as among immigrant populations. Furthermore, it would be beneficial to monitor the receipt of MI on both national and international levels using tools such as barometers or automated research methods (eg, robotic surveys) with comparable measures and study designs. Tracking the evolution of consumers’ access to MI will facilitate more targeted, patient-centered approaches, ultimately guiding consumers and medicine users toward more reliable and accurate MI sources. The incorporation of structured futures methodologies into MI research may further strengthen strategic planning and support systematic anticipation of emerging developments in digital health communication, AI-mediated information environments, and evolving medicine user behaviors [82].

Finally, heterogeneity in sample composition limited interpretability: half of the studies (14/26, 54%) examined heterogeneous medicine user groups, making it difficult to identify the specific medicines used [37-50,60]. This variability also hindered direct comparisons with studies focused on patients with specific medications or chronic conditions. Furthermore, the studies included in the review covered only a small proportion of the overall medicine user population. Five studies did not report the year of data collection [39,43,44,47,58], which may limit the temporal relevance of some findings.

### Conclusions

Despite the increasing role of the internet and social media, traditional sources of MI, such as physicians, pharmacists, and PLs, remain the most commonly used among adult medicine users. Receipt of MI from electronic sources appears more frequent among individuals familiar with the internet, although no clear or consistent temporal increase was identified.

This review is innovative in systematically comparing traditional and electronic MI sources across developed economies during a period of rapid digital transformation. In contrast to much of the existing literature, which has focused on single modalities or treated the internet as a homogeneous source, this study highlights the limited differentiation of digital MI subtypes and the lack of consistent theoretical grounding in current research.

By synthesizing cross-national evidence and identifying methodological and conceptual gaps, this review advances the field by clarifying how traditional and emerging digital MI sources coexist in contemporary health care environments. The findings underscore the need to distinguish between websites, social media platforms, online forums, and AI-mediated tools when examining the receipt of MI and its effects on medication-related perceptions, decisions, and behaviors.

From a practical perspective, these results inform HCPs, policymakers, and digital health developers seeking to optimize reliable, user-centered MI strategies. Addressing digital literacy, misinformation, and equitable access will be increasingly important as algorithmically mediated and AI-based MI tools continue to reshape how medicine users access and act upon MI in real-world settings.

## Supplementary material

10.2196/71984 Multimedia Appendix 1 Search strategies and databases used in the literature review.

10.2196/71984 Multimedia Appendix 2 Criteria of the Mixed Methods Appraisal Tool (MMAT; version 2018).

10.2196/71984 Multimedia Appendix 3 Detailed data extraction table of included studies (n=26).

10.2196/71984 Checklist 1 PRISMA (Preferred Reporting Items for Systematic Reviews and Meta-Analyses) 2020 checklist for systematic reviews.

10.2196/71984 Checklist 2 PRISMA (Preferred Reporting Items for Systematic Reviews and Meta-Analyses) 2020 checklist for abstracts.

10.2196/71984 Checklist 3 PRISMA-S (Preferred Reporting Items for Systematic Reviews and Meta-Analyses–Search extension) checklist for reporting literature searches in systematic reviews.

## Abbreviations

AI: artificial intelligence
EU: European Union
FDA: Food and Drug Administration
HCP: health care professional
MI: medicines information
MMAT: Mixed Methods Appraisal Tool
OTC: over-the-counter
PL: package leaflet
PRISMA: Preferred Reporting Items for Systematic Review and Meta-Analyses
PRISMA-S: Preferred Reporting Items for Systematic Reviews and Meta-Analyses–Search extension
WMI: written medicines information
