# Supplementary material for: Receipt of Medicines Information From the Internet and Other Information Sources Among Adult Medicine Users in Developed Economies, 2010-2025: Systematic Review
Source: J Med Internet Res. 2026 May 20;28:e71984. doi: 10.2196/71984 (PMC13189575; doi:10.2196/71984)
Supplement: Multimedia Appendix 1 [file jmir-v28-e71984-s001.pdf]

Search strategies and databases used in the literature review.

Database	Date of the last search	Search strategy
CINAHL	Jan 15, 2026	TI ("medic* information" OR "drug information") OR AB ("medic* information" OR "drug information") AND TI (seek* OR sought OR search* OR receipt* OR receiv*) OR AB (seek* OR sought OR search* OR receipt* OR receiv*) AND TI (source* OR channel*) OR AB (source* OR channel*) AND TI (patient* OR "medicine user*" OR consumer* OR respondent* OR interviewee* OR women OR men) OR AB (patient* OR "medicine user*" OR consumer* OR respondent* OR interviewee* OR women OR men)
Cochrane Library	Jan 15, 2026	"medic* information" OR "drug information" in Title Abstract Keyword AND seek* OR sought OR search* OR receipt* OR receiv* in Title Abstract Keyword AND source* OR channel* in Title Abstract Keyword AND patient* OR "medicine user*" OR consumer* OR respondent* OR interviewee* OR women OR men in Title Abstract Keyword
ProQuest	Jan 15, 2026	(ti("medic* information" OR "drug information") OR ab("medic* information" OR "drug information")) AND (ti(seek* OR sought OR search* OR receipt* OR receiv*) OR ab(seek* OR sought OR search* OR receipt* OR receiv*)) AND (ti(patient* OR "medicine user*" OR consumer* OR respondent* OR interviewee* OR women OR men) OR ab(patient* OR "medicine user*" OR consumer* OR respondent* OR interviewee* OR women OR men)) AND (ti(source* OR channel*) OR ab(source* OR channel*))
Scopus	Jan 15, 2026	TITLE-ABS-KEY (“medic*information” OR “drug information”) AND TITLE-ABS-KEY (seek* OR sought OR search* OR receipt* OR receiv*) AND TITLE-ABS-KEY (source* OR channel*) AND TITLE-ABS-KEY (patient* OR “medicine user*” OR consumer* OR respondent* OR interviewee* OR women OR men)
Web of Science	Jan 15, 2026	#1 ((TI=("medic* information" OR "drug information")) OR AB=("medic* information" OR "drug information")) OR KP=("medic* information" OR "drug information")<br>#2 ((TI=(seek* OR sought OR search* OR receipt* OR receiv*)) OR AB=(seek* OR sought OR search* OR receipt* OR receiv*)) OR KP=(seek* OR sought OR search* OR receipt* OR receiv*)<br>#3 ((TI=(source* OR channel*)) OR AB=(source* OR channel*)) OR KP=(source* OR channel*)<br>#4 ((TI=(patient* OR "medicine user*" OR consumer* OR respondent* OR interviewee* OR women OR men)) OR AB=(patient* OR "medicine user*" OR consumer* OR respondent* OR interviewee* OR women OR men)) OR KP=(patient* OR "medicine user*" OR consumer* OR respondent* OR interviewee* OR women OR men)<br>#5 (((#1) AND #2) AND #3) AND #4
