# Supplementary material for: Receipt of Medicines Information From the Internet and Other Information Sources Among Adult Medicine Users in Developed Economies, 2010-2025: Systematic Review
Source: J Med Internet Res. 2026 May 20;28:e71984. doi: 10.2196/71984 (PMC13189575; doi:10.2196/71984)
Supplement: Multimedia Appendix 2 [file jmir-v28-e71984-s002.pdf]

Criteria of the Mixed Methods Appraisal Tool (MMAT), version 2018.

Category of study designs	Methodological quality criteria	Responses		
		Yes	No	Can´t tell
Screening questions (for all types)	S1. Are there clear research questions?<br>S2. Do the collected data allow to address the research questions?			
1. Qualitative	1.1. Is the qualitative approach appropriate to answer the research question?<br>1.2. Are the qualitative data collection methods adequate to address the research question?<br>1.3. Are the findings adequately derived from the data?<br>1.4. Is the interpretation of results sufficiently substantiated by data?<br>1.5. Is there coherence between qualitative data sources, collection, analysis and interpretation?			
2. Quantitative randomized controlled trials	2.1. Is randomization appropriately performed?<br>2.2. Are the groups comparable at baseline?<br>2.3. Are there complete outcome data?<br>2.4. Are outcome assessors blinded to the intervention provided?<br>2.5 Did the participants adhere to the assigned intervention?			
3 Quantitative non-randomized	3.1. Are the participants representative of the target population?<br>3.2. Are measurements appropriate regarding both the outcome and intervention (or exposure)?<br>3.3. Are there complete outcome data?<br>3.4. Are the confounders accounted for in the design and analysis?<br>3.5. During the study period, is the intervention administered (or exposure occurred) as intended?			
4. Quantitative descriptive	4.1. Is the sampling strategy relevant to address the research question?<br>4.2. Is the sample representative of the target population?<br>4.3. Are the measurements appropriate?<br>4.4. Is the risk of nonresponse bias low?<br>4.5. Is the statistical analysis appropriate to answer the research question?			
5. Mixed methods	5.1. Is there an adequate rationale for using a mixed methods design to address the research question?<br>5.2. Are the different components of the study effectively integrated to answer the research question?<br>5.3. Are the outputs of the integration of qualitative and quantitative components adequately interpreted?<br>5.4. Are divergences and inconsistencies between quantitative and qualitative results adequately addressed?<br>5.5. Do the different components of the study adhere to the quality criteria of each tradition of the methods involved?			

Question scores:

Yes	1 point
No	0 point
Can´t tell	0 point

Each study is evaluated through two screening questions (S1, S2) and sub-questions depending on the research method used. The sum of the points indicates the total quality of the study. Questions S1 and S2 are excluded from the total score. For mixed methods studies, the overall quality score is the lowest of the study components as the overall quality of a combination cannot exceed the quality of its weakest component.

From: Hong QN, Pluye P, Fàbregues S, Bartlett G, Boardman F, Cargo M, et al. Improving the content validity of the Mixed Methods Appraisal Tool: a modified e-Delphi study. J Clin Epidemiol. 2019;111:49-59. doi: 10.1016/j.jclinepi.2019.03.008
