# Supplementary material for: Receipt of Medicines Information From the Internet and Other Information Sources Among Adult Medicine Users in Developed Economies, 2010-2025: Systematic Review
Source: J Med Internet Res. 2026 May 20;28:e71984. doi: 10.2196/71984 (PMC13189575; doi:10.2196/71984)
Supplement: Multimedia Appendix 3 [file jmir-v28-e71984-s003.pdf]

Detailed data extraction table of included studies (n=26).

STUDIES ON THE RECEIPT OF MI AMONG HETEROGENEOUS MEDICINE USER GROUPS (n=14)

Field	Details
**Surveys, national (n=4)**	
Reference	[38]
Authors	Mononen N, Airaksinen MSA, Hämeen-Anttila K, Helakorpi S, Pohjanoksa-Mäntylä M
Year	2019
Country	Finland
Title	Long-term trends in the receipt of medicines information among adult medicine users: repeated cross-sectional survey in Finland
Setting	National
Study design	Repeated cross-sectional postal survey covering multiple years (1999, 2002, 2005, 2008–2014)
Population/Sample	Medicine users, n=1871 (2010), n=1844 (2011), n=1759 (2012), n=1677 (2013), n=1671 (2014); outpatients; females 63–64%; adults 18-64 years
Recruitment/Sampling	National probability sampling via Population Register Centre of Finland; new independent sample (~5000 individuals) drawn each study year; response rates 66-68% in sample years
Study aim/Research questions	To examine long-term trends in the receipt of MI from different sources among adult medicine users in Finland over multiple years
Description/Measurement of MI receipt	Receipt of MI defined as self-reported receipt of MI used within 12 months from a list of consumer MI sources; measured via survey questions listing MI sources
Outcome measures	Proportions (%) of adult medicine users reporting receipt of MI from each source for each study year; temporal trends across years
Data collection period	Surveys conducted across years annually from 2010–2014
Analysis methods	Descriptive statistical analysis across survey waves; trend assessment of proportions receiving MI from each source across time
Key findings related to MI	• Throughout the study period (2010 to 2014), physicians (51% to 47%), community pharmacists (45% to 46%), and PLs (33% to 34%) were the most common MI sources.<br>• Receipt of MI from the Internet (14% to 16%) remained quite the same.<br>• 26-28% of medicine users reported not receiving MI from any sources listed in the survey, especially among patients using one or two medicines or medicine users without any diagnosed disease.<br>• 38% of medicine users did not report any HCPs as their source of MI in 2014.<br>• Women, people aged ≥45 years, people with ≥3 medicines in use, and people with ≥3 diagnosed diseases received MI more often on their medication than other medicine users.
Theoretical framework	Not reported; descriptive and trend-oriented study
Quality assessment (MMAT[a])	5/5 (high confidence)
Risk of bias	Potential nonresponse bias due to varying response rates over years; recall bias in self-reported MI receipt; sampling might underrepresent older age groups beyond 64 years; representativeness of postal survey may vary over the years
Conflicts/Funding	No conflicting interests reported; funded by Elli Turunen Fund of the Finnish Cultural Foundation
Applicability/Generalizability	Applicable to adult medicine users within the sampled Finnish population; limited generalizability to non-surveyed age groups and to healthcare systems outside similar national contexts; may not generalize to other countries or non-outpatient populations
Notes	Long-term repeated cross-sectional design uniquely captures trends over time; increasing MI from the Internet but persistent dominance of traditional MI sources highlights gradual shifts in consumer information behaviour; subgroup differences may reflect demographic and health status influences
Reference	[39]
Authors	Hämeen-Anttila K, Pietilä K, Pylkkänen L, Pohjanoksa-Mäntylä M
Year	2018
Country	Finland
Title	Internet as a source of medicines information (MI) among frequent Internet users
Setting	National
Study design	Cross-sectional online survey
Population/Sample	Prescription and OTC medicine users, n=2489; outpatients; females 85%; adults ≥18 years
Recruitment/Sampling	Convenience sampling via online distribution by patient organizations, pharmacies authorities, and affiliated email lists
Study aim/Research questions	To explore the Internet as a source of MI compared with other MI sources, identify characteristics of Internet MI users, and describe patterns of Internet use for MI
Description/Measurement of MI receipt	Receipt of MI measured by self-reported use of 14 MI sources; rated on a 4-point Likert scale
Outcome measures	Use of the Internet as an MI source; frequency of use of different MI sources; types of websites used; search strategies and terms; associations between background variables and Internet MI use
Data collection period	January-February 2014
Analysis methods	Descriptive statistics (frequencies and proportions); Pearson's chi-squared tests; multivariable logistic regression
Key findings related to MI	• PLs (90%), pharmacists (83%), and physicians (72%) were more common MI sources than the Internet (68%) among frequent Internet users.<br>• National health portals (56%), search engines (43%), websites of pharmacies (41%), medicines authorities (38%), and commercial health portals (37%) were the most common sources to find MI on the Internet.<br>• Women, people with polytechnic, college, or university degrees, people aged <65 years, daily use of the Internet, and users of vitamins and herbal remedies use the Internet most for receiving MI.
Theoretical framework	Not reported; exploratory descriptive study
Quality assessment (MMAT[a])	4/5 (moderate confidence)
Risk of bias	Selection bias due to convenience online sampling and self-selection into survey; overrepresentation of women, older adults, and highly educated respondents; recall and self-report bias
Conflicts/Funding	No conflicting interests reported; no specific funding declared
Applicability/Generalizability	Applicable to frequent Internet users and medicine users in Finland; limited generalizability to non-Internet users with lower digital literacy
Notes	Study conducted to support development of the Finnish national MI Strategy; highlights the complementary role of the Internet alongside traditional MI sources and the need for guidance toward reliable online information
Reference	[56]
Authors	O'Donovan B, Rodgers RM, Cox AR, Krska J
Year	2019
Country	United Kingdom
Title	Use of information sources regarding medicine side effects among the general population: a cross-sectional survey
Setting	National
Study design	Cross-sectional on-site survey
Population/Sample	Prescription medicine users, n=230 respondents; outpatients; females 61%; adults ≥18 years
Recruitment/Sampling	Convenience sampling; questionnaires distributed by hand to adult pharmacy customers waiting for prescriptions in participating community pharmacies; response rate 25%
Study aim/Research questions	To determine the use and perceived value of MI sources for identifying ADRs and explore associations with coping styles
Description/Measurement of MI receipt	Receipt of MI measured by self-reported predicted and actual use of MI following suspected ADRs
Outcome measures	Predicted and actual use of MI sources; perceived attributes of MI sources (trustworthiness, accessibility); coping style measures (SECope, MBSS)

Data collection period	Not explicitly stated, but distribution included pilot and main data collection prior to December 2019 publication
Analysis methods	Descriptive statistics; chi-squared tests; t-tests and analysis of variance; Spearman correlation; principal component analysis and Cronbach's alpha for SECope; qualitative content analysis of free-text responses
Key findings related to MI	• Physicians (69%), PLs (67%), the Internet (38%), and pharmacists (27%) were the most widely used MI sources for information on ADRs among medicine users.<br>• Usually, people use one (20%), two (31%), or three (33%) information sources to confirm their assessment of the experience as an ADR.
Theoretical framework	Not reported; study incorporates behavioural scales (SECope, MBSS) but no overarching theoretical model described
Quality assessment (MMAT[a])	3/5 (low confidence)
Risk of bias	Selection bias due to pharmacy-based recruitment and voluntary participation; non-response bias; recall bias for past ADRs; overrepresentation of high information-seeking individuals
Conflicts/Funding	No conflicting interests reported; funded by the Medway School of Pharmacy
Applicability/Generalizability	Applicable to adult medicine users in community pharmacy settings in UK; transferability may be limited to populations with similar access to pharmacies and WMI
Notes	First study exploring MI source use for medicine ADRs in relation to coping styles; highlights discrepancies between perceived trustworthiness and actual use of MI sources
Reference	[61]
Authors	Santos B, Blondon KS, Van Gessel E, Cerutti B, Backes C, Locher S, Guignard B, Bonnabry P, Carpenter D, Schneider MP
Year	2022
Country	Switzerland
Title	Patients' perceptions of conflicting information on chronic medications: a prospective survey in Switzerland
Setting	National
Study design	Cross-sectional online or on-site survey
Population/Sample	Prescription medicines users, n=405; inpatients and outpatients; females 57%; adults ≥18 years
Recruitment/Sampling	Consecutive recruitment in one academic community pharmacy, three partner community pharmacies, and three inpatient medical units at Geneva University Hospitals; surveys administered in person
Study aim/Research questions	To investigate patients' perceptions of conflicting information on chronic medications, and understand its impact on self-management and healthcare system navigation
Description/Measurement of MI receipt	Receipt of MI assessed via patients' self-reported perception of conflicting MI during the past 12 months using a 5-point response scale; sources of MI were also recorded
Outcome measures	Prevalence of receipt conflicting MI; types and sources of conflicting MI; consequences for medication adherence and healthcare navigation; associations with sociodemographic and clinical variables
Data collection period	March-December 2019
Analysis methods	Descriptive statistics; chi-squared tests; multivariate logistic regression assessing associations with sociodemographic and clinical factors
Key findings related to MI	• 47% received conflicting information related to one or more medication topics, most commonly on side effects (22%) and duration of treatment (18%).<br>• General practitioners (82%), special physicians (74%), and pharmacists (49%) were the most common HCPs involved in conflicting information.<br>• Younger (18-50 years) people received conflicting information from the Internet and social media more often than older (>50 years)<br>• Patients who received conflicting information were prescribed more medicines than those who did not receive conflicting information.
Theoretical framework	Not reported; descriptive epidemiological study
Quality assessment (MMAT[a])	3/5 (low confidence)
Risk of bias	Selection bias due to voluntary participation; limited inpatient recruitment; reliance on patient perceptions rather than objectively verified conflicting MI; potential recall bias; missing data due to non-mandatory survey items
Conflicts/Funding	No conflicting interests reported; funded by Federal Office of Public Health (FOPH), Interprofessional Health Promotion Program
Applicability/Generalizability	Applicable to chronic disease patients in Swiss outpatient healthcare settings; generalizability may be limited to similar healthcare systems and patients with multiple healthcare contacts
Notes	First Swiss study to quantify the received of conflicting MI in chronic patients; survey specifically developed and pretested; findings highlight the need for improved interprofessional communication and consistency of MI
**Surveys, local (n=6)**	
Reference	[52]
Authors	Krska J, Morecroft CW
Year	2013
Country	United Kingdom
Title	Informing patients about medicines: a hospital in-patient survey in England
Setting	Hospitals (n=6) in North West England
Study design	Cross-sectional on-site survey
Population/Sample	Hospital inpatients using prescription medicines, n=1218; females 51%; adults ≥18 years
Recruitment/Sampling	Convenience sampling; questionniares distributed via ward to patiens in hospitals
Study aim/Research questions	To determine how often hospital inpatients receive MI, and whether this varies by patient characteristics or hospital
Description/Measurement of MI receipt	Measured via three closed-ended questions adapted from NICE audit tool: (1 receipt of verbal information about medicines, (2 receipt of WMI, (3 discussion of medicine-related concerns
Outcome measures	Self-reported provision of verbal MI, WMI, and discussion of concerns regarding medicines
Data collection period	January 2011
Analysis methods	Descriptive statistics; chi-squared tests for associations between outcomes and demographic/hospital variables; Spearman's correlation coefficients for co-relationships between outcomes
Key findings related to MI	• 44% of the hospital patients had been fully informed about their medicines by a hospital physician, nurse, or pharmacist during their hospital stay, 34% were partly informed, and 20% of the patients had not received any information.<br>• 19% of the patients had received WMI during their admission, 12% had received PLs, and 8% had received other WMI. 83% of patients who received WMI had read it.<br>• 80% of the patients said they had not received any WMI while in hospital or could not recall receiving any.<br>• 18% of the patients were given written and verbal information, with 60 % being given verbal information only and 1% being given WMI only. 21% of patients did not recall being given either WMI or verbal information.
Theoretical framework	Not reported
Quality assessment (MMAT[a])	4/5 (moderate confidence)
Risk of bias	Recall bias (self-reported data); selection bias (ward staff identified eligible patients); no validation via observation or clinician report
Conflicts/Funding	No conflicting interests reported; no specific funding declared
Applicability/Generalizability	Applicable to adult medical inpatients in NHS hospitals; generalizability to other countries, non-medical wards, or non-English speakers limited
Notes	Highlights inconsistency in receiving MI and patient engagement; high response rate
Reference	[53]
Authors	Krska J, Morecroft CW
Year	2013
Country	United Kingdom
Title	Patients' use of information about medicine side effects in relation to experiences of suspected adverse drug reactions:

	a cross-sectional survey in medical in-patients
Setting	Hospitals (n=6) in North West England
Study design	Cross-sectional on-site survey
Population/Sample	Hospital inpatients using prescription medicines, n=1218; females 51%, adults ≥18 years
Recruitment/Sampling	Convenience sampling; questionniares distributed via ward to patiens in hospitals
Study aim/Research questions	To explore how patients use PLs and other MI sources, and whether use differs with experience of suspected ADRs
Description/Measurement of MI receipt	Self-reported use of of PLs; seeking additional MI, perceived knowledge of medicine risks
Outcome measures	Frequency of reading PL; seeking information about ADRs; associations with experience/exposure (demographics, medicines)
Data collection period	Not explicitly reported; likely conducted prior to publication (2011–2012)
Analysis methods	Descriptive statistics; Chi-squared and Fisher's exact tests for associations; binary logistic regression for factors associated with experiencing ADRs; quantitative analysis of open-ended responses using pre-defined categoriescomparisons between groups based on ADR experience and receipt of MI
Key findings related to MI	• 74% of the hospital patients usually read the PL supplied with their medicines.<br>• 42% of the patients always read the PL for all medicines, but only 33% read the PL only for new medicines.<br>• 19% of the patients never read the PL, whilst 7% of the patients read the PL only when something unexpected happens.<br>• 56% of the patients never sought more information about possible ADRs.<br>• The patients searched for additional information from HCPs (51%) or the Internet (29%).
Theoretical framework	Not reported
Quality assessment (MMAT[a])	4/5 (moderate confidence)
Risk of bias	Self-report/recall bias by patients; selection bias (English-speaking, inpatient medical wards only); social desirability bias; potential non-response bias (numbers of patients excluded or refusing not reported)
Conflicts/Funding	No conflicting interests reported; funded by Liverpool John Moores University
Applicability/Generalizability	Generalizable to hospital medical inpatients in England; may not generalize to surgical patients, non-English speakers, or outpatients
Notes	PL use and MI-seeking is common but not universal; majority of patients value MI on ADRs and feel knowledgeable; educational level and prior medicines experience influence the receipt of MI and perception of ADRs
Reference	[45]
Authors	Cooper JM, Garrett T
Year	2014
Country	Australia
Title	Providing medicines information and education to hospital in-patients: patients' experiences and preferences
Setting	Hospitals (n=2) in New South Wales
Study design	Cross-sectional on-site survey
Population/Sample	Hospital discharged inpatients using prescription medicines, n=292; females 61%; adults ≥18 years
Recruitment/Sampling	Convenience sampling at hospital discharge for eligible patients; response rate 22%
Study aim/Research questions	To explore patients' experiences and expectations on the receipt of MI during hospital admission
Description/Measurement of MI receipt	Patients self-report of having received MI or counselling during admission or at discharge
Outcome measures	Recall of receiving MI; preferred provider of MI; contenet of counselling
Data collection period	Not explicitly reported; data collected across designated hospital discharge period (likely months within 2014)
Analysis methods	Descriptive statistics (frequencies, proportions) summarizing survey responses
Key findings related to MI	• 53% of hospital patients recalled being seen by a pharmacist during their in-patient stay or before discharge. 81% of these patients indicated that the pharmacist explained the purpose of the medication and how to take or use it to 76% of patients. 53% of these patients did not recall being told about side effects.<br>• 15% of patients indicated they wanted to speak to someone about their medications but did not get the opportunity while in the hospital. Paradoxically, 29% of these had already responded that a pharmacist had seen them during their current admission.
Theoretical framework	None reported
Quality assessment (MMAT[a])	3/5 (low confidence)
Risk of bias	Low response rate (22%); recall bias; selections bias; self-repoert data; limited representatives
Conflicts/Funding	No conflicting interests reported; no specific funding declared
Applicability/Generalizability	Applicable to adult hospital inpatients discharged with new medications in Australian teaching hospitals; limited generalizability to other settings or patient groups
Notes	Patients value MI but often prefer physician over pharmacists as MI providers; findings highlight unmet needs for counselling time and the importance of post-discharge continuity of medicines education
Reference	[54]
Authors	DeLorme DE, Huh J, Reid LN
Year	2011
Country	United States
Title	Source selection in prescription drug information seeking and influencing factors: applying the comprehensive model of information seeking in an American context
Setting	Community-based sample; Southeastern metropolitan area
Study design	Cross-sectional computer-assisted telephone survey
Population/Sample	Prescription medicine users, n=234; outpatients; females 71%; adults ≥18 years
Recruitment/Sampling	Random digit dialing from commercially available prescreened telephone samples; telephone CATI interviews conducted by trained interviews; response rate 32%
Study aim/Research questions	To investigate the extent and direction of prescription MI receiving, identify MI sources used, and examine how demographic and health-related factors influence source selection using a modified Comprehensive Model of Information Seeking (CMIS)
Description/Measurement of MI receipt	Prescription MI sources were measured using: 1) self-reported pre- and post-physician visit MI receiving, 2) open-ended recall of MI sources used, 3) perceived usefulness of 20 different prescription MI sources
Outcome measures	Extent of prescription MI-seeking (none; pre- or post-visit; both pre- and post-visit), 2) perceived usefulness of different MI source types, and 3) actual use of different prescription MI sources
Data collection period	Not reported
Analysis methods	Descriptive statistics, ANOVA, logistic regression, correlation analysis, and path analysis using the multiple regression technique (testing the modified CMIS)
Key findings related to MI	• Most popular MI sources on prescription medicines were health-related websites (24%), websites on specific brands of medicines (22%), pharmacists (21%), physicians (21%), the Internet (18%), and PLs (12%).<br>• The average MI sources per person was 1.7, and the maximum was 5.
Theoretical framework	Modified Comprehensive Model of Information Seeking (CMIS), based on the health belief model, uses and gratifications theory, and media exposure/appraisal model
Quality assessment (MMAT[a])	2/5 (very low confidence)
Risk of bias	Low response rate; regional sample; gender overrepresentation (women); self-report bias; use of mostly single-item measures; cross-sectional design limits causal inference
Conflicts/Funding	No conflicting interests reported; no specific funding declared
Applicability/Generalizability	Generalizable primarily to adults in a southeastern US metropolitan area who use prescription medicines; caution is required for national or international generalization due to regional sample and demographic imbalances
Notes	MI-seeking was limited in extent; Internet sources played a prominent role; findings suggest contextual limitations of CMIS and need for model refinement; highlights potential digital divide in prescription MI access
Reference	[57]
Authors	Perry D, Librizzi B, Ngu L, Ricciardello M, Street A, Clifford R, Goodman C, Peeling P, Salter SM
Year	2020

Country	Australia
Title	Medication information and supply behaviours in elite and developing athletes
Setting	State-based sporting institute in Perth
Study design	Cross-sectional online survey
Population/Sample	Elite and developing athletes using prescription or non-prescription medicines, n=90; outpatients, adults ≥18 years
Recruitment/Sampling	Convenience sampling; athletes attending the institute´s gymnasium were invited to participate in person
Study aim/Research questions	To investigate the receipt of MI among athletes, and to identify the role of pharmacists in athlete care
Description/Measurement of MI receipt	Self-reported frequency and trust of MI sources over the past 6 months; survey included Likert scales and free-text responses
Outcome measures	Sources and frequency of medication supply; use and trust of MI sources; pharmacy use for minor ailments; knowledge of the institute's medication policy; perceptions of the pharmacist's role
Data collection period	August 2017
Analysis methods	Descriptive statistics, chi-square/Fisher's exact tests, ANOVA, post-hoc t-tests with Bonferroni correction; qualitative inductive analysis of free-text comments
Key findings related to MI	• Pharmacists were the most common MI source among athletes using prescription medicines (74%) or non-prescription medicines (71%).<br>• 63% of athletes 'sometimes' consulted pharmacists for MI.<br>• Athletes using medicines seek MI more often on the Australian Sports Anti-Doping Authority (ASADA) and World Anti-Doping Agency (WADA) websites than pharmacists for MI. 63% of athletes always seek MI from the ASADA website.
Theoretical framework	Not reported
Quality assessment (MMAT[a])	2/5 (very low confidence)
Risk of bias	Convenience sample; recall bias; self-reported bias; selection bias; social desirability bias
Conflicts/Funding	No conflicting interests reported; funded by the University of Western Australia
Applicability/Generalizability	Applicable to adult elite and developing athletes in similar Australian institutional sport settings; limited generalizability to adolescents or non-institutional athletes
Notes	First comprehensive Australian study on athlete MI behaviours and pharmacist role; highlights gaps in policy knowledge and underutilisation of pharmacists for MI
Reference	[62]
Authors	Bergmo TS, Sandsdalen V, Manskow US, Småbrekke L, Waaseth M.
Year	2023
Country	Norway
Title	Internet use for obtaining medicine information: cross-sectional survey
Setting	Online (social media) and community pharmacies (n=11) in Tromsø
Study design	Cross-sectional online/paper-based survey
Population/Sample	Prescription and OTC medicine users, n=303; outpatients, females 65%
Recruitment/Sampling	Convenience sampling; in-person recruitment at community pharmacies and supplementary recruitment via social media
Study aim/Research questions	To explore the receipt of MI from the Internet among pharmacy customers, identify factors associated with Internet use, describe use of other MI, and assess trust in different sources and websites
Description/Measurement of MI receipt	Self-reported receipt of MI from various sources over the past 12 months, measured via a structured questionnaire; Internet use defined by selecting the Internet as an information source
Outcome measures	Proportion using different MI sources; extent of Internet use; reasons for seeking MI; level of trust in MI sources, and specific websites
Data collection period	September-October 2020
Analysis methods	Descriptive statistics; chi-square tests; t test or Mann–Whitney test; multivariable logistic regression with backward selection to identify factors associated with Internet use
Key findings related to MI	• 41% of medicine users reported using the Internet as a source of MI.<br>• HCPs were the most trusted MI source, with 91% of respondents expressing trust. In contrast, approximately half of Internet users trusted online information, particularly content from national health portals and official health authority websites.<br>• Women were more likely than men to receive MI from pharmacists and PLs. Among women, those younger than 65 years more frequently used pharmacists, PLs, and the Internet compared to women aged 65 years or older. Additionally, individuals with regular prescriptions were more likely to receive MI from physicians, pharmacists, and PLs than those without regular prescriptions.<br>• Age was the only factor significantly associated with Internet use.<br>• Most medicine users sought MI from 1-3 different sources, while 14% reported using more than three sources.
Theoretical framework	Not reported
Quality assessment (MMAT[a])	1/5
Risk of bias	Selection bias; self-report and recall bias; possible overrepresentation of respondents interested in MI
Conflicts/Funding	No conflicting interests reported; funded by the Gravitate-Health project
Applicability/Generalizability	Findings applicable to adult pharmacy customers in similar Norwegian or comparable high-income settings; limited generalizability to populations not using community pharmacies or with limited Internet access
Notes	First Norwegian study examining Internet use for MI among pharmacy customers; highlights continued reliance on HCPs alongside the growing use of trusted official web-based sources
**Qualitative interviews (n=2)**	
Reference	[49]
Authors	Haverhals LM, Lee CA, Siek KA, Darr CA, Linnebur SA, Ruscin JM, Ross SE
Year	2011
Country	United States
Title	Older adults with multi-morbidity: medication management processes and design implications for personal health applications
Setting	Community and clinic settings in Denver and Boulder, Colorado: an academic hospital-based ambulatory geriatric clinic, a municipal senior center, an independent-living residential facility, and an assisted/independent-living facility
Study design	Semi-structured individual and group interviews
Population/Sample	Prescription medicine users, n=32; outpatients, females 10%; older adults ≥65 years
Recruitment/Sampling	Purposive sampling; recruitment via flyers, in-person visits, word of mouth
Study aim/Research questions	To explore medication management processes among adults with multimorbidity
Description/Measurement of MI receipt	Receipt of MI was explored qualitatively through interviews and focus groups; participants described their MI sources and how they sought, evaluated, and used this information in self-management
Outcome measures	Themes regarding medication self-management challenges: seeking reliable MI, maintaining autonomy, worrying about polypharmacy, reconciling discrepancies between conventional and alternative therapies, tracking and coordinating health information among providers
Data collection period	Not reported
Analysis methods	Thematic analysis using deductive and inductive coding; investigator triangulation; iterative team review; field notes, photographs, transcripts
Key findings related to MI	• Older adults (≥65 years) most often received MI from pharmacists and physicians, followed by PLs and the Internet.<br>• Challenges included evaluating the credibility of MI, coordinating multiple sources, and communicating with providers.
Theoretical framework	Not reported; exploratory qualitative approach
Quality assessment (MMAT[a])	5/5 (high confidence)
Risk of bias	Small sample size, purposive sampling, geographic limitation, and inclusion of caregiver dyads; investigator triangulation strengthens credibility
Conflicts/Funding	No conflicting interests reported; funded by the Robert Wood Johnson Foundation Project Health Design Grant
Applicability/Generalizability	Applicable to community-dwelling older adults with multimorbidity in similar US urban settings; limited generalizability

	due to small, purposively selected sample
Notes	Study conducted as part of a larger project to develop a prototype interoperable personal health application tailored to older adults' medication self-management needs; a strong multidisciplinary research team and an iterative qualitative design proces
Reference	[58]
Authors	Tong V, Raynor DK, Aslani P
Year	2018
Country	Australia and United Kingdom
Title	Receipt and use of spoken and written over-the-counter medicine information: insights into Australian and UK consumers' experiences
Setting	Community pharmacies in Sydney (Australia) and Leeds (UK)
Study design	Semi-structured individual interviews
Population/Sample	OTC medicine users, n=37 (Australia) and n=39 (UK); outpatients; females 50%; adults ≥18 years
Recruitment/Sampling	Purposive sampling; Australia: flyers, online advertisements, market research company; UK: consumer database from Luto Research
Study aim/Research questions	To explore consumers´ receipt and use of spoken MI and WMI and the role of PLs in self-management
Description/Measurement of MI receipt	Self-reported recall of spoken MI received at point of purchase and WMI received and/or used following the most recent OTC purchase
Outcome measures	Receipt of spoken MI and WMI; use and rereading of OTC labels/leaflets; perceived role and value of WMI; MI-seeking behaviors
Data collection period	April 2013 - April 2014
Analysis methods	Thematic analysis of verbatim transcripts; matrices to compare within and between Australian and UK cohorts; independent verification by second researcher
Key findings related to MI	• The majority of Australian and UK consumers reported that no spoken MI was actively provided when they purchased their OTC medicine. Instructions for the use of medicine were the most often information received.<br>• OTC medicine users tended not to read labels/PLs if they were familiar with the medicine.<br>• When WMI was read, instructions for the use of medicine were commonly read at home.<br>• Most Australian consumers reported that they did not receive a PL with their OTC medicine. In contrast, the overwhelming majority of UK consumers noted receiving a PL. Specifically, UK consumers commented that PL was standard with OTC medicine packages.
Theoretical framework	Not reported
Quality assessment (MMAT[a])	5/5 (high confidence)
Risk of bias	Recall bias; self-selection of participants; health literacy not screened; qualitative design limits generalizability
Conflicts/Funding	One author (DKR) reported being cofounder of Luto Research; one author (DKR) reported receive travel grant from Royston George Booker Scholarship and University of Sydney Grant-in-Aid
Applicability/Generalizability	Transferable insights into OTC information behaviors in community pharmacy contexts in Australia and the UK; not statistically generalisable due to qualitative design and purposive sampling
Notes	Majority of purchases were repeat purchases; differences in PLs regulation between Australia (not mandatory for all OTC medicines) and UK (mandatory PLs) did not substantially change usage patterns; highlights importance of improving OTC information delivery strategies to enhance medication safety

Mixed methods (n=2)

Reference	[55]
Authors	Mackridge AJ, Rodgers R, Lee D, Morecroft CW, Krska J
Year	2018
Country	United Kingdom
Title	Cross-sectional survey of patients' need for information and support with medicines after discharge from hospital
Setting	Hospitals (n=6) in North West England
Study design	Mixed-methods; two-stage study: 1) face-to-face cross-sectional inpatient questionnaire at discharge, 2) telephone follow-up survey post-discharge
Population/Sample	Hospital-discharged inpatients using prescription medicines, n=444 (study 1) and n=99 (study 2); females 53% (study 1) and 45% (study 2); adults ≥18 years
Recruitment/Sampling	Convenience sampling; patients identified through screening by ward pharmacists/nurses
Study aim/Research question	To explore the receipt of information about medication changes during admission and post-discharge support among hopital patients
Definition/Measurement of MI receipt	Receipt of MI measured via self-reported understanding of medicine changes, discussions with HCPs, and perceived sufficiency of information both during admission and post-discharge
Outcome measures	Sources and type of MI received; awareness and understanding of medicine changes; perceived involvement in decisions; post-discharge support needs and experiences; occurrence of medicine-related problems post-discharge
Data collection period	January-March 2015
Analysis methods	Descriptive statistics and frequencies; Chi-squared tests; Spearman's correlation coefficient; independent coding of qualitative follow-up responses
Key findings related to MI	• Study 1: 51% of hospital-discharged patients recalled discussing their medicines with one HCP during their hospital stay, 30% discussed their medication with more than one HCP, and 16% did not recall discussing them with any HCP. Information about medication changes was most frequently provided by consultant medical staff (39%), with pharmacists providing information least often (17%). 37% of patients recalled being offered information before a medicine was prescribed.<br>• Study 2: 5% of the patients had spoken to a pharmacist on medication changes, although 35% reported drug-related problems (DRPs) after being discharged.
Theoretical framework	Not reported
Quality assessment (MMAT[a])	4/5 (moderate confidence)
Risk of bias	Self-reporting bias; convenience sampling; study 2 follow-up included 22% of initial participants; hospitals anonymized to reduce bias
Conflicts/Funding	No conflicting interests reported; no specific funding declared
Applicability/Generalizability	Applicable to adult medical inpatients in UK hospitals; ation changes at discharge; generalizable to similar healthcare settings with ward-based pharmacy services, though variability across hospitals may limit broader applicability
Notes	Study included both pre- and post-discharge data to reduce recall bias; highlights underutilization of pharmacists in post-discharge support; emphasizes importance of improving patient involvement and MI receiving during hospital stays
Reference	[41]
Authors	Bastholm-Rahmner P, Gustafsson LL, Aggefors K, Ateva K, Elfving S, Eriksen J, Jirlow M, Juhasz-Haverinen M, Malmström RE, Nikpour-Ardaly M, Röjvall M, Vallin M, Andersén-Karlsson E, Ovesjö ML
Year	2018
Country	Sweden
Title	Patients' knowledge and attitudes to the Wise List - a drug formulary from the Stockholm Drug and Therapeutic committee
Setting	Primary healthcare centers (n=4) in Stockholm
Study design	Mixed-methods; study 1: on-site survey (included in this review), study 2[b]: focus group discussions (not included in this review)
Population/Sample	Study 1: Medicine users, n=312; outpatients; females 59%, adults 18–96 years
Recruitment/Sampling	Study 1: Patients approached in waiting rooms at four purposively selected primary healthcare centers
Study aim/Research question	To explore the receipt of MI and knowledge, need and attitudes regarding the list of recommended essential medicines (Wise List)
Definition/Measurement of MI receipt	Patient-reported knowledge and use of the Wise List and sources/preferences for MI
Outcome measures	Study 1: recognition and use of the Wise List; perceived usefulness/benefit (1–10), ability to find needed MI, willingness to recommend, awareness of producer, and general MI-seeking (sources, desire for MI)
Data collection period	Study 1: February 2015
Analysis methods	Study 1: descriptive analysis

Key findings related to MI	• Study 1: 63% of the medicine users had searched for MI. Physicians (36%) and the Internet (31%) were adult medicine users' most common MI sources.
Theoretical framework	Not reported
Quality assessment (MMAT[a])	3/5 (low confidence)
Risk of bias	Study 1: Selection bias; social desirability; limited primary healthcare centers
Conflicts/Funding	No conflicting interests reported; partly funded by Karolinska Institutet and Stockholm Healthcare Region
Applicability/Generalizability	Applicable to similar primary care settings in Stockholm and potentially to contexts aiming to communicate evidence-based, non-commercial MI to patients; may require adaptation for other regions or healthcare systems
Notes	Highlights importance of patient involvement in developing MI tools; patient-tailored and accessible presentation of formulary MI is critical; study influenced revisions to the Wise List patient version

STUDIES ON THE RECEIPT OF MI AMONG ADULTS USING SPECIFIC MEDICINES (n=12)

Field	Details
**Surveys, national (n=4)**	
Reference	[40]
Authors	Amundsen S, Øvrebø TG, Amble NMS, Poole AC, Nordeng H
Year	2016
Country	Norway
Title	Use of antimigraine medications and information needs during pregnancy and breastfeeding: a cross-sectional study among 401 Norwegian women
Setting	National
Study design	Cross-sectional online survey
Population/Sample	Pregnant and breastfeeding women using antimigraine medicines, n=401; outpatients, females 100%, adults ≥18 years
Recruitment/Sampling	Self-selected participants recruited through advertisements on Norwegian pregnancy/motherhood websites, the Norwegian Migraine Association website, Facebook, posters/flyers in primary healthcare units, and a maternity outpatient clinic
Study aim/Research question	To explore the use of antimigraine medications, the receipt of MI and MI needs during pregnancy and breastfeeding
Definition/Measurement of MI receipt	Receipt of MI was measured by self-reported perceived need for medicines safety information before, during, and after pregnancy; MI sources used; experience of conflicting MI; trust in specific MI sources
Outcome measures	Receipt of MI and need of MI; use of antimigraine medication during pregnancy; patterns of acute and preventive medication use before, during, and after pregnancy; migraine severity (MIGSEV scale); experience of conflicting MI and related medication discontinuation
Data collection period	October 2013 -February 2014
Analysis methods	Descriptive statistics; Pearson chi-square/Fisher´s exact tests; Cronbach's alpha for MIGSEV; uni-/multivariable logistic regression
Key findings related to MI	• 72% of pregnant women sought safety information about their antimigraine medications.<br>• Physicians (87%), the Internet (47%), PLs (42%), midwives (29%), and pharmacists (19%) were the most frequently consulted MI sources among pregnant women.<br>• Mean number of consulted MI sources was 1.8.<br>• 45% of pregnant women who consulted multiple MI sources had experienced conflicting information.<br>• 39% of pregnant women had discontinued the use of the medicine in question because of conflicting MI.
Theoretical framework	Not reported; epidemiological and pharmacoepidemiological approach
Quality assessment (MMAT[a])	5/5 (high confidence)
Risk of bias	Self-selection bias; no calculable response rate; recall bias; potential differential recall between pregnant women and mothers; overrepresentation of highly educated and possibly more resourceful women; possible selection of women with more severe migraine
Conflicts/Funding	One author (ACP) reported lectures/advisory boards for several pharma companies (1999–2014); funded by Northern Norway Regional Health Authority
Applicability/Generalizability	Applicable to Norwegian pregnant and breastfeeding women with migraine and similar high-income settings with high Internet access; generalizability limited by web-based recruitment and higher educational level of participants
Notes	Comprehensive assessment of medication use before, during, and after pregnancy; highlights high MI and impact of conflicting medicine safety information on adherence; severe migraine strongly predicted medication use during pregnancy
Reference	[54]
Authors	Leonardo N, Lester S, Graham M, Barrett C, Whittle S, Rowett D, Buchbinder R, Hill CL
Year	2020
Country	Australia
Title	Selection and perception of methotrexate treatment information in people with rheumatoid arthritis
Setting	National
Study design	Cross-sectional online survey
Population/Sample	Rheumatoid arthritis patients using/have used methotrexate, n=742; outpatients; females 76%; adults 56-60 years
Recruitment/Sampling	Email invitation via national rheumatology registry Australian Rheumatology Association Database, ARAD; online survey distributed via email to registered rheumatoid arthritis patients; response rate 80%
Study aim/Research question	To explore the receipt of MI on methotrexate among rheumatoid arthritis patients
Definition/Measurement of MI receipt	Receipt of MI was measured by self-reported consultation of specific methotrexate MI sources; participants rated the perceived valence of MI from each source; influence on beliefs was assessed using methotrexate-specific Beliefs about Medicines Questionnaire (BMQ) scores
Outcome measures	Number and type of MI sources used; perceived positive/negative nature of MI; general and methotrexate-specific BMQ scores; current vs prior methotrexate use
Data collection period	October-November2017
Analysis methods	Descriptive statistics; Chi-square tests and t-tests; Multivariable Poisson regression; seemingly unrelated regression models
Key findings related to MI	• Rheumatologists (98%), general practitioners (55%), the Internet (39%), educational websites (38%), and pharmacists (37%) were the most common MI sources of methotrexate among rheumatoid arthritis patients.<br>• 26% of the rheumatoid arthritis patients reported rheumatologists as their only MI source.<br>• The median number of MI sources consulted was 3 (mean 1-5 sources).
Theoretical framework	Not reported
Quality assessment (MMAT[a])	5/5 (high confidence)
Risk of bias	Cross-sectional design; recall bias; registry-based sample; responders older and more likely on biologics than non-responders; potential selection bias
Conflicts/Funding	No conflicting interests reported; ARAD funded by unrestricted educational grants (various pharmaceutical companies) and prior NHMRC Enabling Grant, one author (RB) supported by NHMRC Senior Principal Research Fellowship
Applicability/Generalizability	Applicable to Australian rheumatoid arthritis patients registered in ARAD; may generalize to similar high-income healthcare systems with specialist-led rheumatoid arthritis management; limited representativeness of patients not enrolled in national registries
Notes	High response rate strengthens internal validity; findings informed development of Australian National Prescribing Service (NPS) educational materials for consumers and education programs for physicians and pharmacists regarding safe and effective methotrexate use; highlights strong influence of rheumatologists and structured educational websites on patient medication beliefs
Reference	[59]
Authors	Otón T, Carmona L, Andreu JL
Year	2022
Country	Spain

Title	What do patients on methotrexate need and expect at the clinic? An online patient survey
Setting	National
Study design	Cross-sectional online survey
Population/Sample	Immune-mediated disease patients using methotrexate, n=283; outpatients, females 82%; adults ≥18 years
Recruitment/Sampling	Primary recruitment via Twitter; snowball sampling allowed
Study aim/Research question	To investigate the experience, needs, and expectations of patients on methotrexate, including the receipt of MI, shared decision-making, and quality of patient-physician communication
Definition/Measurement of MI receipt	Receipt of MI measured via self-reported survey items assessing: whether WMI was provided during consultation; usefulness of provided materials; whether websites were recommended by physicians; usefulness of suggested websites; reading and perceived usefulness of PL; satisfaction with communication and shared decision-making; preferences for format of MI
Outcome measures	Receipt, usefulness, and format preference of MI; participation in shared decision-making; perceived support from HCPs and family; impact of methotrexate on daily life and ADRs
Data collection period	March-July 2020
Analysis methods	Descriptive statistics; no imputation for missing data
Key findings related to MI	• 80% of immune-mediate disease patients reported that they read the PLs.<br>• 15% of patients reported that WMI had been offered at the physician consultation.<br>• 12% of patients had been pinpointed to websites by their physicians.<br>• 80% of patients reported not receiving any information during the physician consultation.
Theoretical framework	Not reported
Quality assessment (MMAT[a])	2/5 (very low confidence)
Risk of bias	Selection bias; self-selection bias; self-reported data; possible overrepresentation of certain diseases; cross-sectional design; limited control of data quality due to online design
Conflicts/Funding	No conflicting interests reported; no specific funding declared
Applicability/Generalizability	Applicable to Spanish adults with immune-mediated diseases using methotrexate; limited generalizability to other countries, languages, or recruitment methods; likely more representative of digitally engaged patients
Notes	High response rate; majority female, aged 31–60; oral vs. subcutaneous methotrexate roughly equal; patients reported unmet needs for structured, accessible and reliable MI; findings could inform strategies for patient education and shared decision-making
**Surveys, local (n=6)**	
Reference	[42]
Authors	Bults M, Beaujean DJMA, Wijkmans CJ, Timen A, Richardus JH, Voeten HACM
Year	2012
Country	The Netherlands
Title	Why did patients with cardiovascular disease in the Netherlands accept Q fever vaccination?
Setting	Municipal public health service 's-Hertogenbosch
Study design	Questionnaire-based individual interviews
Population/Sample	Cardiovascular medicine users, n=413; outpatients; females 39%; 70% aged > 60 years
Recruitment/Sampling	All patients (n=500) vaccinated on selected data collection days were invited to participate; convenience sample based on vaccination attendance; ~ 80% response rate
Study aim/Research question	To examine reasons for accepting Q fever vaccination, including risk perception, feelings of doubt, social influence, MI-seeking behavior, preventive measures, and perceptions of received MI and governmental action among high-risk cardiovascular patients
Definition/Measurement of MI receipt	Receipt of MI and communication processes were measured through self-reported exposure to MI sources, perceived amount, comprehensiveness, and reliability of MI, satisfaction with communication, and involvement of social networks in decision-making
Outcome measures	Reasons for vaccination acceptance; perceived severity and vulnerability; feelings of doubt; social influence; MI-seeking behavior; preventive measures taken; satisfaction with received MI; perceptions of governmental action
Data collection period	January-April 2011
Analysis methods	Descriptive statistics; chi-squared tests for group differences by gender, age, education, and employment status; multivariable regression analyses for outcomes with multiple significant predictors
Key findings related to MI	• General physicians (60%), family (32%), specialists (13%), and friends (13%) were the most frequently reported MI sources about Q fever vaccination among cardiovascular patients.<br>• 30% of patients had actively sought information about Q fever vaccination.<br>• Patients aged 60 years or younger (39%), those with a higher educational level (42%), and those employed (41%) sought information more often.
Theoretical framework	Integrated health behavior model drawing on Protection Motivation Theory (PMT) and the Health Belief Model (HBM)
Quality assessment (MMAT[a])	5/5 (high confidence)
Risk of bias	Selection bias; social desirability bias; cross-sectional design; no data on vaccine decliners
Conflicts/Funding	No conflicting interests reported; funded by the Netherlands Organization for Health Research and Development
Applicability/Generalizability	Applicable to high-risk cardiovascular patients in Q fever outbreak settings; limited generalizability to other populations, countries, non-accepters of vaccination; results particularly relevant for vaccination campaigns requiring targeted risk communication
Notes	High response rate and immediate post-vaccination data collection reduced recall bias; study provides rare empirical insight into vaccination acceptance during a Q fever outbreak and supports tailored risk communication strategies
Reference	[59]
Authors	Houser SH, Au DW, Miller MJ, Chen L, Outman RC, Ray MN, Saag KG, Weech-Maldonado R
Year	2016
Country	United States
Title	Socio-demographic differences in risk information seeking sources for non-steroidal anti-inflammatory drugs (NSAIDS)
Setting	Primary care clinics (n=39) in Alabama
Study design	Cross-sectional computer-assisted telephone survey
Population/Sample	Prescription NSAID or OTC medicine (ibuprofen/naproxen) users, n=220; outpatients; females 75%; adults ≥19 years
Recruitment/Sampling	Convenience sampling; eligibility determined by patient exit cards at physician practices; participation rate 69%
Study aim/Research question	To identify primary sources of NSAID risk information, and associations between socio-demographic factors and choice of MI source
Definition/Measurement of MI receipt	Receipt of MI measured via the question: "Who/what is your main source of information about problems or risks associated with taking prescription NSAIDs?"
Outcome measures	Primary NSAID risk information source used; associations of MI source with socio-demographic characteristics
Data collection period	March-November 2011
Analysis methods	Descriptive statistics; multivariable multinomial logistic regression
Key findings related to MI	• Physicians (57%), the Internet (17%), and pharmacists (16%) were the primary sources of NSAID risk information.<br>• Compared to patients who use the Internet as a primary source of NSAID risk information, patients who were Black/African-American and 65 years of age or older were more likely to use a physician.<br>• Older patients were more likely to use pharmacists than the Internet as an MI source.<br>• Women were less likely to use the pharmacist than the Internet as an MI source.
Theoretical framework	Not reported
Quality assessment (MMAT[a])	5/5 (high confidence)
Risk of bias	Selection bias; self-report bias; cross-sectional design; geographic limitation
Conflicts/Funding	No conflicting interests reported; funded by AHRQ Deep South Musculoskeletal Center for Education and Research in Therapeutics and NIA Deep South Resource Center on Minority Aging (RCMAR)
Applicability/Generalizability	Applicable to similar primary care populations in the US and may inform clinicians on demographic differences in NSAID risk information seeking; cautious generalization to other regions or health care systems due to cultural/infrastructure differences
Notes	Highlights socio-demographic disparities in use of Internet vs. HCPs for medication risk information, emphasizes importance of

	tailored communication strategies for age, race, and gender differences in patient MI-seeking behavior; suggests interventions at provider-patient and community levels to enhance equitable access to reliable MI
Reference	[47]
Authors	deToro J, Cea-Calvo L, Battle E, Carmona L, Arteaga MJ, Fernández S, González CM
Year	2019
Country	Spain
Title	Perceptions of patients with rheumatic diseases treated with subcutaneous biologicals on their level of information: RHEU-LIFE survey
Setting	Outpatient rheumatology clinics across Spanish hospitals (n=50)
Study design	Cross-sectional on-site survey
Population/Sample	Arthritis medicines (s.c. biological) users, n=592; outpatients; females 58%; adults ≥18 years
Recruitment/Sampling	Consecutive sampling; 20 eligible patients per hospital invited by rheumatologists; response rate 59%
Study aim/Research question	To investigate the receipt of MI about biological medicines among arthritis patients
Definition/Measurement of MI receipt	Patients self-reported the level of MI received via an ad hoc survey developed by rheumatologists and patients
Outcome measures	Sources of MI; satisfaction with MI received in hospital; training in self-administration of biologic; perceived adequacy of information on treatment characteristics and ADRs
Data collection period	September-October 2015
Analysis methods	Descriptive statistics; Chi-squared and Fisher's exact tests
Key findings related to MI	• Rheumatologists (75%), primary care physicians (22%), and nurses (19%) were the principal sources of subcutaneous (s.c.) biological medicines among arthritis patients.<br>• 45% of arthritis patients sought information about biological medicines from other MI sources than the hospital's HCPs.<br>• 45% of arthritis patients received oral MI and WMI from biological medicines, 46% oral MI only, and 6% WMI only.<br>• 9% of patients stated that they had not been taught to inject the biological medicines.
Theoretical framework	Not reported
Quality assessment (MMAT[a])	4/5 (moderate confidence)
Risk of bias	Selection bias; recall bias; nonresponse bias; social desirability
Conflicts/Funding	Some authors employees of MSD, others received honoraria or consultancy fees from pharmaceutical companies; funded by Merck Sharp & Dohme
Applicability/Generalizability	Applicable to adult Spanish patients with rheumatoid arthritis, axial spondyloarthritis, or psoriatic arthritis receiving subcutaneous biologicals in outpatient hospital settings; generalizability limited to similar healthcare contexts
Notes	Survey developed ad hoc with patient input; high patient satisfaction linked to rheumatologist involvement; emphasizes importance of guidance toward reliable MI sources
Reference	[56]
Authors	Geryk LL, Blalock S, DeVellis RF, Morella K, Carpenter DM
Year	2016
Country	United States
Title	Associations between patient characteristics and the amount of arthritis medication information patients receive
Setting	Community and hospital-based
Study design	Cross-sectional online survey
Population/Sample	Arthritis medicines users, n=328; outpatients; females 79%; adults ≥18 years
Recruitment/Sampling	Hospital system mailings and general recruitment via patient websites, clinics, support groups, and media advertisements
Study aim/Research question	To explore the receipt of MI among arthritis patients
Definition/Measurement of MI receipt	Self-reported amount of arthritis medication information received from 15 MI sources
Outcome measures	Amount of arthritis medication information received (mean score), by individual MI sources and source categories
Data collection period	May 2010-January 2011
Analysis methods	Descriptive statistics; bivariate analyses; multivariable linear regression
Key findings related to MI	• 99% of arthritis patients received MI from at least one source when prescribed a new arthritis medicine.<br>• Physicians, PLs, the Internet, and brochures were the most common MI sources.<br>• 2% of arthritis patients indicated that they did not receive MI from physicians, 33% did not receive MI from pharmacists, and 49% did not receive MI from nurses.<br>• Greater receipt of MI was associated with greater medication adherence, taking more medications, greater medication-taking concerns, more satisfaction with physician medication-related support, and Black compared to White race.
Theoretical framework	Not reported
Quality assessment (MMAT[a])	4/5 (moderate confidence)
Risk of bias	Self-reported data; selection bias; low response; limited representativeness
Conflicts/Funding	No conflicting interests reported; funded by the National Center for Research Resources
Applicability/Generalizability	Applicable to US adults with Internet access and diagnosed osteoarthritis or rheumatoid arthritis; limited generalizability to more diverse or clinically severe populations
Notes	First study to examine patient characteristics associated with arthritis medication information receipt across multiple sources; includes both solicited and unsolicited information; highlights role of HCPs and media in information delivery
Reference	[51]
Authors	Carpenter DM, DeVellis RF, Hogan SL, Fisher EB, DeVellis BM, Jordan JM
Year	2011
Country	United States
Title	Use and perceived credibility of medication information sources for patients with a rare illness: differences by gender
Setting	Primarily Chapel Hill, North Carolina
Study design	Cross-sectional online survey
Population/Sample	Vasculitis medicine users, n=232; outpatients; females 69%; adults 21-82 years
Recruitment/Sampling	Recruitment through vasculitis patient conference, conferences, research networks, clinics, support groups, websites, and newsletters
Study aim/Research question	To examine the use and perceived credibility of MI sources among vasculitis patients
Definition/Measurement of MI receipt	Receipt of MI was measured by self-reported frequency of obtaining vasculitis MI from 12 sources over the past year
Outcome measures	Frequency of use of MI sources, and perceived credibility of MI sources
Data collection period	Not reported
Analysis methods	Descriptive statistics; multivariate analysis of covariance; follow-up contrasts for each MI source; independent t-tests
Key findings related to MI	• Physicians, the Internet, and PLs were the most frequently used MI sources among vasculitis patients, both men and women.<br>• Women receive MI from PLs and the Internet more often than men.<br>• Men receive MI more often from their spouse, partner, or nurse.<br>• Physicians, the Internet, pharmacists, and other vasculitis patients were perceived as the most credible sources of MI for both genders.
Theoretical framework	Not reported
Quality assessment (MMAT[a])	3/5 (low confidence)
Risk of bias	Selection bias; recall bias; potential nonresponse bias; low response rate
Conflicts/Funding	No conflicting interests reported; funded by Renal Epidemiology Predoctoral Traineeship, Thurston Arthritis Research Center Postdoctoral Fellowship, ACR REF/Abbott Health Professional Graduate Medical Student Research Preceptorship
Applicability/Generalizability	Applicable to English-speaking, Internet-using adults with vasculitis; not generalizable to non-Internet users, more diverse populations, or patients with other diseases without caution

Notes	First study describing medication-specific information-seeking in vasculitis; highlights importance of physicians and Internet in rare disease contexts; suggests involving spouse/partner in male patients' care; rare disease context likely limits usefulness of family/friends as information sources
Reference	[52]
Authors	Carpenter DM, Elstad EA, Blalock SJ, DeVellis RF
Year	2014
Country	United States
Title	Conflicting medication information: prevalence, sources, and relationship to medication adherence
Setting	Primarily University of North Carolina Hospital System and community sources
Study design	Cross-sectional online survey
Population/Sample	Arthritis medicine users, n=328; outpatients; females 79%; adults ≥18 years
Recruitment/Sampling	Mailings from a university hospital system; additional recruitment via clinics, arthritis support groups, local media, Craigslist and prior research participants
Study aim/Research question	To examine prevalence and sources of conflicting MI among arthritis patients associations with sociodemographic/clinical factors and medication adherence
Definition/Measurement of MI receipt	Conflicting MI defined as contradictory information about medication topics from two or more sources; measured using a 12-item self-report scale assessing frequency of conflicting MI across medication topics
Outcome measures	Conflicting MI score; medication adherence; MI source use; perceived regimen complexity and disease severity
Data collection period	May 2010-January 2011
Analysis methods	Descriptive statistics; factor analysis and internal consistency testing; bivariate analyses; multivariable linear regression controlling for demographic and clinical covariates
Key findings related to MI	• Physicians, PLs, the Internet, and brochures and pamphlets were the most common MI sources among arthritis patients.<br>• 80% of arthritis patients have received conflicting MI about at least one medication topic.<br>• Other physicians (27%), the media, such as TV, radio, magazines, newspapers (22%), and the Internet (21%) were the most common sources of conflicting MI.<br>• More significant amounts of conflicting MI were associated with non-White race, more significant perceived medication regimen complexity, higher perceived disease severity, more MI sources used, and worse medication adherence.
Theoretical framework	Chronic Care Model and Information-Motivation-Behavioral Skills Model
Quality assessment (MMAT[a])	3/5 (low confidence)
Risk of bias	Self-reported measures; cross-sectional design (no causal inference); recall bias; selection bias; limited demographic diversity
Conflicts/Funding	No conflicting interests reported; funded by the Thurston Arthritis Research Center Postdoctoral Fellowship, Novartis Pharmaceuticals, ACR REF/Abbott Health Professional Graduate Student Research Preceptorship, and the U.S. National Center for Research Resources
Applicability/Generalizability	Applicable to US arthritis patients with Internet access and long-term medication use; limited generalizability to more diverse, newly diagnosed, or non-Internet-using populations
Notes	First study to comprehensively document sources of conflicting MI in arthritis patients; developed and psychometrically evaluated a 12-item conflicting information scale; conflicting MI was prevalent but modest in intensity; effect size on adherence small but statistically significant; highlights complexity of MI-seeking
**Qualitative interviews (n=3)**	
Reference	[63]
Authors	Bergsholm YKR, Feiring M, Charnock C, Holm LB, Krogstad T
Year	2023
Country	Norway
Title	Exploring patients' adherence to antibiotics by understanding their health knowledge and relational communication in encounters with pharmacists and physicians
Setting	Community pharmacies and general practice in central urban areas of the South-Eastern Norwegian health region
Study design	Focus group interview
Population/Sample	Antibiotic medication users, n=8; outpatients; gender not reported; adults ≥18 years
Recruitment/Sampling	Open invitations (acquaintances); recruitment poster with QR code at pharmacy, social media (Facebook)
Study aim/Research question	To explore the receipt of MI among antibiotic users
Definition/Measurement of MI receipt	Receipt of MI explored qualitatively through focus group discussions examining: what MI received, from which MI sources, how MI was communicated, and how patients understood and used MI; no quantitative measurement; based on thematic analysis of narratives
Outcome measures	Thematic findings related to: patient knowledge about antibiotics and antimicrobial resistance, sources of MI, relational communication, and perceived influence of communication on adherence
Data collection period	October 2020-January 2021
Analysis methods	Reflexive systematic text condensation; manual coding with collaborative analysis by multidisciplinary team; theoretically informed interpretation
Key findings related to MI	• Antibiotic medicine users actively sought MI from digital platforms (e.g., HealthNorway), printed material, and face-to-face encounters.<br>• Patients were more receptive to MI at the pharmacy after physician consultation.<br>• Digital information increased empowerment but required health literacy to interpret correctly.<br>• Relational communication (patient-centered dialogue, open-ended questions, tailored advice) improved understanding and motivation for adherence.<br>• Time pressure, lack of privacy, and standardized "autopilot" counseling reduced effectiveness.
Theoretical framework	Not reported
Quality assessment (MMAT[a])	5/5 (high confidence)
Risk of bias	Selection bias; urban sample; gender imbalance; some participants previously acquainted; focus group setting may limit expression of dissenting views
Conflicts/Funding	No conflicting interests reported; funded by The Foundation for the Promotion of Norwegian Pharmacy
Applicability/Generalizability	Applicable to similar high-income primary care settings with community pharmacy systems; limited generalizability to rural areas, other healthcare systems, or quantitatively measurable adherence outcomes
Notes	Emphasizes relational communication as central to adherence; highlights pharmacist's role as final healthcare contact before treatment initiation; identifies misconceptions about AMRantimicrobial resistance as barrier to optimal antibiotic use; suggests potential benefit of open-ended questions and tailored counseling strategies
Reference	[50]
Authors	Hayden C, Neame R, Tarrant C
Year	2015
Country	United Kingdom
Title	Patients' adherence-related beliefs about methotrexate: a qualitative study of the role of written patient information
Setting	Rheumatology outpatient department at the University Hospitals of Leicester NHS Trust, East Midlands
Study design	Semi-structured individual interviews
Population/Sample	Arthritis patients using oral methotrexate, n=15; outpatients; females 73%; adults ≥18 years
Recruitment/Sampling	Purposive sampling from outpatient clinics and hospital monitoring databases; patients approached in clinic or contacted by post
Study aim/Research question	To explore the receipt of MI and adherence-related beliefs about methotrexate among arthritis patients
Definition/Measurement of MI receipt	Receipt of MI was explored qualitatively through patients' accounts of exposure to written PILs, verbal MI from physicians, and informal MI sources such as other patients and the Internet
Outcome measures	Patient experiences and beliefs regarding methotrexate adherence, necessity beliefs, concerns, and recepit of MI

Data collection period	November 2013-January 2014
Analysis methods	Thematic analysis combining inductive and deductive approaches; analysis informed by the necessity–concerns framework but grounded in participant data; patients used MI-seeking and avoidance strategies to manage dissonance between necessity and concerns
Key findings related to MI	• All arthritis patients reported discussions with physicians about their diagnosis and medication, and each patient recalled being given WMI.<br>• WMI helped a little in reinforcing expectations of benefit or alleviating concern.<br>• Arthritis patients reported that PLs lacked details about how the medicine works and, specifically, the nature of the benefits that they could reasonably expect.<br>• Verbal information from physicians was highly valued but focused on disease progression rather than medication efficacy or effectiveness.<br>• Receipt of MI from the Internet often helped little to allay concerns and resolve dissonance.
Theoretical framework	Necessity–Concerns Framework and Cognitive dissonance theory
Quality assessment (MMAT[a])	5/5 (high confidence)
Risk of bias	Selection bias; recall bias; exclusion of patients who refused or discontinued methotrexate; no participants over 80 years of age
Conflicts/Funding	No conflicting interests reported; funded by the University of Leicester intercalated Bachelor of Science fund
Applicability/Generalizability	Applicable to adults with inflammatory arthritis starting methotrexate in UK secondary care settings; limited transferability to other healthcare systems or patients who discontinue treatment
Notes	Highlights tensions between safety-focused WMI and patients' need for reassurance about effectiveness; identifies MI-seeking and avoidance as coping strategies for managing ambivalence and dissonance
Reference	[60]
Authors	Wakob I, Wintsche I, Frisch A, Remane Y, Laufs U, Bertsche T, Schiek S
Year	2022
Country	Germany
Title	Assessment of patients' views on drug benefits and risks: an interview study with cardiovascular patients
Setting	Cardiology department of a tertiary-care University hospital in Leipzig
Study design	Semi-structured individual interviews
Population/Sample	Cardiovascular medicine users, n=102; inpatients; females 35%; adults 21-91 years
Recruitment/Sampling	Consecutive sampling; eligible patients admitted to the cardiology ward
Study aim/Research question	To explore the benefits and risks of medication and the receipt of MI among cardiovascular patients
Definition/Measurement of MI receipt	Receipt of MI was assessed through patient self-report during interviews, including perceived benefits and risks of cardiovascular medicines and MI sources
Outcome measures	MI sources and desire for additional information; general benefit-risk estimation; types and frequency of mentioned benefits; whether benefits motivate adherence; types and frequency of mentioned risks; whether risks were perceived as bothersome, concerning, or led to nonadherence; PHQ-4 score
Data collection period	July-October 2018
Analysis methods	Descriptive statistics; chi-square tests; Mann–Whitney U tests; PHQ-4 scores; categorization using ATC, ICD-10, and CTCAE classifications
Key findings related to MI	• Physicians (92%), pharmacists (27%), PLs (26%), and the Internet (19%) were the most common MI sources on the benefits of medicines among cardiovascular patients.<br>• Physicians (45%), PLs (36%), relatives and friends (29%), pharmacists (14%), and the Internet (14%) were the most common MI sources on the risks of medicines among cardiovascular patients.
Theoretical framework	Not reported
Quality assessment (MMAT[a])	5/5 (high confidence)
Risk of bias	Selection bias; response bias; recall bias; no objective adherence measurement; interviewer not blinded
Conflicts/Funding	No conflicting interests reported; funded by the budgets of the participating departments with institutional support for open-acc publishing
Applicability/Generalizability	Applicable to adult cardiovascular inpatients in tertiary-care hospital settings in Germany; limited generalizability to outpatients or other healthcare systems
Notes	Strong distinction between surrogate markers and clinical benefits; PHQ-4 useful for identifying patients vulnerable to risk concerns; highlights need for balanced, individualized MI to support adherence

[a]Mixed Methods Appraisal Tool (MMAT), version 2018; Score: 5=100% compliance with all quality evaluation criteria (high confidence), 4=80% of quality evaluation criteria met (moderate confidence), 3=60% of quality evaluation criteria met (low confidence), 2=40% of quality evaluation criteria met (very low confidence), 1=20% of quality evaluation criteria met, 0=0% of quality evaluation criteria met, [b]Receipt of MI was not studied in study 2
