# Supplementary material for: Receipt of Medicines Information From the Internet and Other Information Sources Among Adult Medicine Users in Developed Economies, 2010-2025: Systematic Review
Source: J Med Internet Res. 2026 May 20;28:e71984. doi: 10.2196/71984 (PMC13189575; doi:10.2196/71984)
Supplement: Checklist 3 [file jmir-v28-e71984-s006.pdf]

PRISMA-S Checklist for reporting literature searches in systematic reviews.

Section/topic	#	Checklist item	Location(s) Reported
**INFORMATION SOURCES AND METHODS**			
Database name	1	Name each individual database searched, stating the platform for each.	Page 4
Multi-database searching	2	If databases were searched simultaneously on a single platform, state the name of the platform, listing all of the databases searched.	-
Study registries	3	List any study registries searched.	-
Online resources and browsing	4	Describe any online or print source purposefully searched or browsed (e.g., tables of contents, print conference proceedings, web sites), and how this was done.	-
Citation searching	5	Indicate whether cited references or citing references were examined, and describe any methods used for locating cited/citing references (e.g., browsing reference lists, using a citation index, setting up email alerts for references citing included studies).	Page, Figure 1
Contacts	6	Indicate whether additional studies or data were sought by contacting authors, experts, manufacturers, or others.	-
Other methods	7	Describe any additional information sources or search methods used.	Page 6, Figure 1
**SEARCH STRATEGIES**			
Full search strategies	8	Include the search strategies for each database and information source, copied and pasted exactly as run.	Appendix 4
Limits and restrictions	9	Specify that no limits were used, or describe any limits or restrictions applied to a search (e.g., date or time period, language, study design) and provide justification for their use.	Page 4-6, Table 1
Search filters	10	Indicate whether published search filters were used (as originally designed or modified), and if so, cite the filter(s) used.	Page 5-6, Table 1, Appendix 3
Prior work	11	Indicate when search strategies from other literature reviews were adapted or reused for a substantive part or all of the search, citing the previous review(s).	-
Updates	12	Report the methods used to update the search(es) (e.g., rerunning searches, email alerts).	-
Dates of searches	13	For each search strategy, provide the date when the last search occurred.	Appendix 4
**PEER REVIEW**			
Peer review	14	Describe any search peer review process.	Page 4
**MANAGING RECORDS**			
Total Records	15	Document the total number of records identified from each database and other information sources.	Figure 1
Deduplication	16	Describe the processes and any software used to deduplicate records from multiple database searches and other information sources.	Page 6, Figure 1

From: Rethlefsen ML, Kirtley S, Waffenschmidt S, Ayala AP, Moher D, Page MJ, et al. PRISMA-S: an extension to the PRISMA statement for reporting literature searches in systematic reviews. J Med Libr Assoc. 2021;109(2):174-200.doi: 10.5195/jmla.2021.962
